# Elastic, Dynamic Viscoelastic and Model-Derived Fibril-Reinforced Poroelastic Mechanical Properties of Normal and Osteoarthritic Human Femoral Condyle Cartilage

**DOI:** 10.1007/s10439-021-02838-4

**Published:** 2021-08-02

**Authors:** Mohammadhossein Ebrahimi, Mikko A. J. Finnilä, Aleksandra Turkiewicz, Martin Englund, Simo Saarakkala, Rami K. Korhonen, Petri Tanska

**Affiliations:** 1 Department of Applied Physics, University of Eastern Finland, POB 1627, 70211 Kuopio, Finland; 2 Research Unit of Medical Imaging, Physics and Technology, Faculty of Medicine, University of Oulu, Oulu, Finland; 3 Clinical Epidemiology Unit, Department of Clinical Sciences Lund, Orthopaedics, Faculty of Medicine, Lund University, Lund, Sweden

**Keywords:** Articular cartilage, Osteoarthritis, Mechanical testing, Mechanical properties, Finite element analysis, OARSI grading

## Abstract

Osteoarthritis (OA) degrades articular cartilage and weakens its function. Modern fibril-reinforced poroelastic (FRPE) computational models can distinguish the mechanical properties of main cartilage constituents, namely collagen, proteoglycans, and fluid, thus, they can precisely characterize the complex mechanical behavior of the tissue. However, these properties are not known for human femoral condyle cartilage. Therefore, we aimed to characterize them from human subjects undergoing knee replacement and from deceased donors without known OA. Multi-step stress-relaxation measurements coupled with sample-specific finite element analyses were conducted to obtain the FRPE material properties. Samples were graded using OARSI scoring to determine the severity of histopathological cartilage degradation. The results suggest that alterations in the FRPE properties are not evident in the moderate stages of cartilage degradation (OARSI 2-3) as compared with normal tissue (OARSI 0-1). Drastic deterioration of the FRPE properties was observed in severely degraded cartilage (OARSI 4). We also found that the FRPE properties of femoral condyle cartilage related to the collagen network (initial fibril-network modulus) and proteoglycan matrix (non-fibrillar matrix modulus) were greater compared to tibial and patellar cartilage in OA. These findings may inform cartilage tissue-engineering efforts and help to improve the accuracy of cartilage representations in computational knee joint models.

**Supplementary Information:**

The online version contains supplementary material available at 10.1007/s10439-021-02838-4.

## Introduction

Mechanical properties of articular cartilage play a vital role in the knee by enabling the proper function of the joint. Those properties originate from the multi-phasic heterogeneous structure of the extracellular matrix, which comprises of interstitial fluid, collagen fibrils, and negatively charged proteoglycans (PGs). These constituents and their interactions determine the highly nonlinear mechanical response of articular cartilage.^9^,^25^ The PGs are the main regulator of the cartilage mechanical behavior when the interstitial fluid flow has seized.^3^,^47^ On the other hand, both the collagen fiber network and pressurized interstitial fluid control the mechanical behavior of cartilage during transient and cyclic loads.^34^,^43^

Osteoarthritis (OA) is known to have detrimental effects on articular cartilage structure and function during the early stages of the disease. For instance, the PG loss is progressive and that reduces the cartilage load-bearing capacity in equilibrium.^23^ Either at the same time or subsequently, the collagen network fibrillates, and part of the tissue-swelling related pretension of the fibrils is lost. All these changes also increase the hydraulic permeability of the tissue which diminishes the interstitial fluid pressurization and weakens the transient response of cartilage.^7^,^16^,^30^

The direct measurement of the mechanical properties of cartilage constituents is not possible. However, this is possible by combining an experimental measurement with computational approaches, such as finite element method-based material modeling. For example, fibril-reinforced poroelastic (FRPE) material modeling has been reported to be able to capture the complex dynamic, transient and equilibrium mechanical responses of articular cartilage.^14^,^24^,^35^ The main advantage of this material model (and its derivations) is its capability to link the mechanical responses to the mechanical material properties of the main cartilage constituents. These constituent-specific mechanical material properties have earlier been characterized for human tibia,^9^ patellar^23^ and hip joint^36^ cartilage. Moreover, MRI-based studies showed more severe morphological changes and higher OA grades in femoral cartilage compared to tibial and patellar cartilages in early OA,^18^,^48^ while another study showed *vice-versa*.^8^ The mechanical environment and strain levels experienced by femoral cartilage are however different than the confronting tibial and patellar cartilages.^11^,^49^ Femoral cartilage might be adapted to different loading environment, which causes its properties to differ from those of tibial or patellar cartilage. This might also affect OA initiation and progression, for instance, different properties might contribute to different lesion progression in different cartilage sites.^38^ Therefore, characterizing the cartilage mechanical properties in a site-specific manner helps to understand how the tissue function differs in each site. While earlier studies have characterized the elastic and dynamic viscoelastic properties of human femoral condyle cartilage,^4^,^31^ the constituent-specific FRPE mechanical material properties remain still uncharted.

Constituent-specific mechanical material properties of normal and OA femoral condyle cartilage would be useful in the future computational knee joint modeling studies, which largely rely on material properties obtained from animals.^17^,^29^ In addition, the biomimicry of tissue-engineered cartilage should be assessed against normal tissue to promote the functional restoration of damaged cartilage.^5^ Therefore, a comprehensive investigation of the constituent-specific properties of the native human femoral condyle cartilage could also benefit tissue engineering efforts.

Thus, the primary aim of this study was to characterize the FRPE material properties of normal and osteoarthritic human femoral condyle cartilage and compare them to the cartilage properties from other sites in the knee and hip (from previous studies). The secondary aim was to evaluate how the elastic, dynamic viscoelastic and FRPE material properties of human femoral condyle cartilage differ in various stages of OA. Our previous study showed that the PG matrix- and collagen fibril network-related properties of human tibial cartilage substantially deteriorate at the moderate stage of OA.^9^ Therefore, we hypothesize that these same properties of femoral condyle cartilage are also weakened at the moderate stages of OA, while the tissue permeability remains unchanged until late OA. To evaluate our hypothesis, we perform experimental multi-step stress-relaxation and sinusoidal measurements in indentation geometry and optimize the FRPE properties by finding the best match between experimental and model-derived data.

## Materials and Methods

### Sample Preparation

Thirty-five osteochondral samples from medial and lateral femoral condyle of 14 total knee replacement (TKR) patients and 10 healthy donors were obtained. The study was approved by the regional ethical review board of Lund University (Dnr 2015/39 and Dnr 2016/865). See the supplementary material for details on sample preparation.

### Indentation Testing, Elastic and Dynamic Viscoelastic Properties

**Figure 1:**
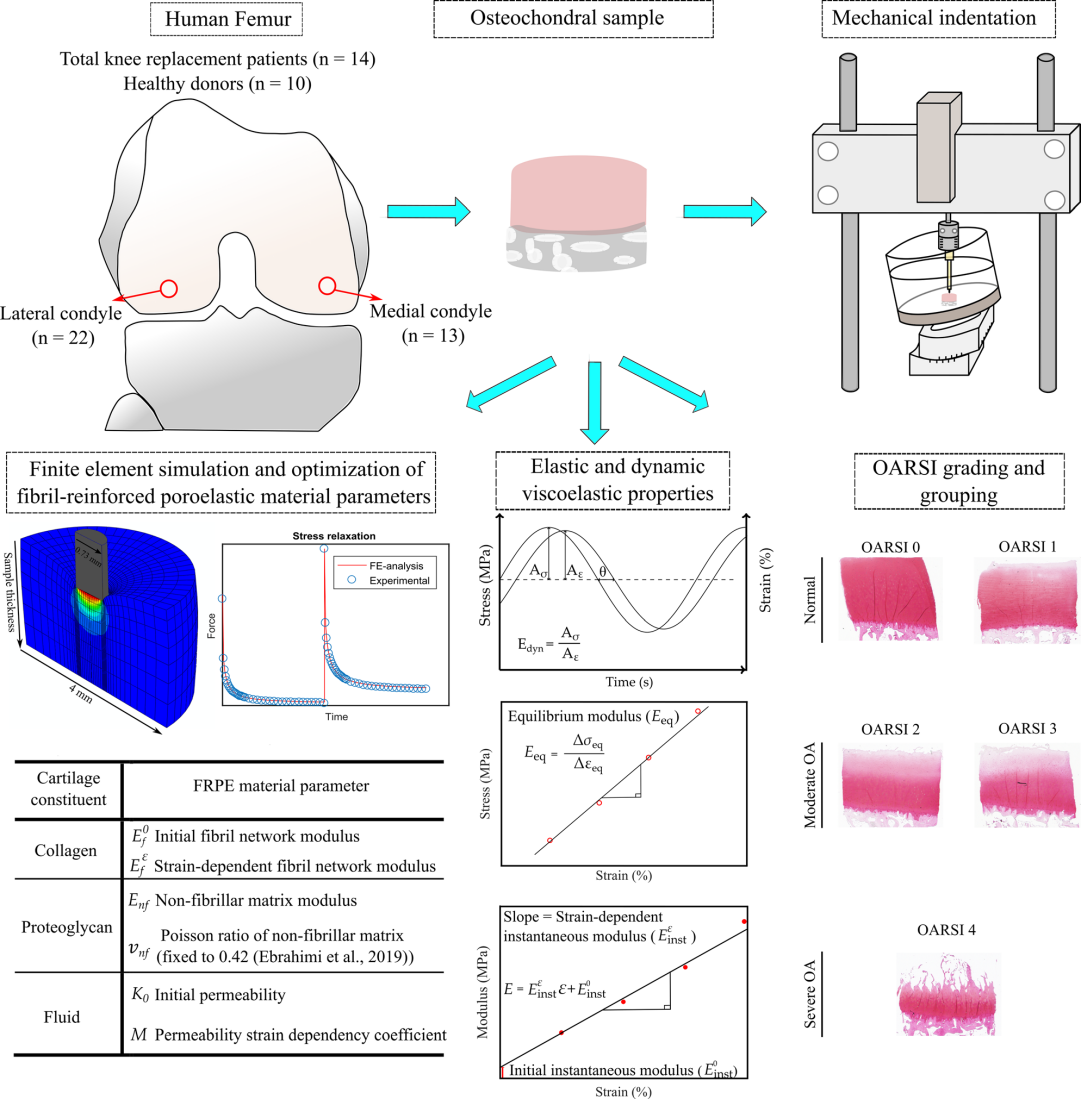
The workflow of the study. Osteochondral samples were prepared from human femoral condyles and mechanically tested in an indentation geometry. First, elastic and dynamic viscoelastic properties were extracted from the stress-relaxation and dynamic testing data (Hayes correction was then applied). Then, sample-specific fibril-reinforced poroelastic (FRPE) finite element models of cartilage were constructed and their mechanical responses were fitted to the experimental data to obtain FRPE material parameters (figure depicts only the 2nd and 3rd steps of the stress-relaxation data that was used in the optimization routine). Afterward, the histopathological OA state of the samples (i.e. OARSI grade) was quantified from Safranin-O stained histological sections.

First, a pre-stress of 12.5 kPa followed by a 4-step stress relaxation test was applied. Each step consisted of 5% strain and 15 min relaxation time. Following the stress-relaxation, a dynamic sinusoidal test using 2% strain amplitude with frequencies of 0.005, 0.05, 0.1, 0.25, 0.5, 0.625, 0.833 and 1 Hz was conducted. Elastic properties of cartilage were calculated from the stress-relaxation curves: the equilibrium modulus (${E}_{\mathrm{eq}}$) was calculated from a slope of a linear least-squares fit to the stress-strain points at the equilibrium (Figure 1, middle column), while the initial instantaneous modulus (intercept, ${E}_{\mathrm{inst}}^{0}$,) and the strain-dependent instantaneous modulus of cartilage (slope, ${E}_{\mathrm{inst}}^{\upvarepsilon }$) were calculated from a linear least-squares fit to the instantaneous moduli measured at each stress-relaxation step. Dynamic viscoelastic properties were calculated from dynamic sinusoidal test curves for each frequency: the dynamic modulus (${E}_{\mathrm{dynamic}}^{\mathrm{i Hz}}$) was defined as the ratio of the stress and strain amplitudes in the dynamic test, while the phase angle (${\theta }_{\mathrm{dynamic}}^{\mathrm{i Hz}}$) was extracted from the Fourier transform of dynamic data and the phase difference was calculated by subtracting the displacement and force phase angles. See the supplementary material for details.

### Finite Element Analysis and Optimization

Sample-specific axisymmetric finite element models were created and cartilage tissue was modeled using the FRPE material model. In this material model, articular cartilage is composed of an elastic fibrillar matrix (representing the collagen fiber network) and a porous hyperelastic non-fibrillar matrix (representing the PG matrix), filled with fluid. The total stress tensor ${{\varvec{\sigma}}}_{\mathrm{t}}$ is defined as the stress caused by fibrillar and non-fibrillar matrices in addition to (pore) fluid pressure:^9^

1 $${{\varvec{\sigma}}}_{\mathrm{t}}={{\varvec{\sigma}}}_{\mathrm{nf}}+{{\varvec{\sigma}}}_{\mathrm{f}}-p\mathbf{I},$$

where ${{\varvec{\sigma}}}_{\mathrm{nf}}$ and ${{\varvec{\sigma}}}_{\mathrm{f}}$ are the stress tensors of the non-fibrillar and fibrillar matrices, respectively, $\mathbf{I}$ is the unit tensor and *p* is the fluid pressure. The FRPE material parameters (${E}_{\mathrm{f}}^{0}$ the initial fibril network modulus, ${E}_{\mathrm{f}}^{\upvarepsilon }$ the strain-dependent fibril network modulus, ${E}_{\mathrm{nf}}$ the non-fibrillar matrix modulus, ${k}_{0}$ the initial permeability, $M$ the permeability strain-dependency coefficient)^9^ were obtained by minimizing the difference between the simulation results and experiments in the 2nd and 3rd stress-relaxation steps. See the supplementary material for details on the material model, finite element analysis, optimization and FRPE material properties (see also Figure 1, left column).

### OA Grading and Samples Grouping

After the mechanical indentation experiments, the samples (i.e. the same samples used in the indentation tests) were fixed in formalin, dehydrated in graded alcohol solutions, embedded in paraffin, and stained with Safranin-O. These histological sections were then semi-quantitatively graded using the Osteoarthritis Research Society International (OARSI) histopathology grading system^40^ to determine the severity of OA (Figure 1, right column). Three experts assessed the samples independently and assigned the OARSI grade to each sample. The final grade was a consensus of their opinion. Samples were also pooled based on the main OARSI grade to different OA progression groups namely (1) the *normal* group which had intact cartilage surface (*n* = 17, OARSI grades 0–1), (2) the *moderate OA* group which had fibrillation and minor abrasion of the most superficial cartilage (*n* = 15, OARSI grades 2–3), and (3) the *severe OA* group which had fissures, cartilage erosion and cartilage matrix loss in the superficial zone (*n* = 3, OARSI grades 4).

### Statistical Analysis

We provide descriptive data on all the parameters per OARSI grade and compartment in the Supplementary Tables S1, S4, S6 and S8. An age and BMI adjusted linear mixed-effects (LME) model was used to analyze the dependent variables (i.e. the FRPE (${E}_{\mathrm{f}}^{0}$, ${E}_{\mathrm{f}}^{\upvarepsilon }$, ${E}_{\mathrm{nf}}$,${ k}_{0}$ and $M$) as well as elastic (${E}_{\mathrm{eq}},$ ${E}_{\mathrm{inst}}^{0}$ and ${E}_{\mathrm{inst}}^{\upvarepsilon }$) and dynamic viscoelastic (${E}_{\mathrm{dynamic}}^{\mathrm{i Hz}}$ and ${\theta }_{\mathrm{dynamic}}^{\mathrm{i Hz}}$) material parameters) and compare mean values between the groups. Statistical analyses were performed in Stata v15 and IBM SPSS Statistics (version 25, IBM Corporation, Armonk, NY, USA). See the supplementary material for details. Results are presented as box plots with minimum and maximum whiskers unless otherwise stated.

## Results

### Model-Derived Constituent-Specific Fibril-Reinforced Poroelastic Properties of Normal and OA Femoral Condyle Cartilage

**Figure 2:**
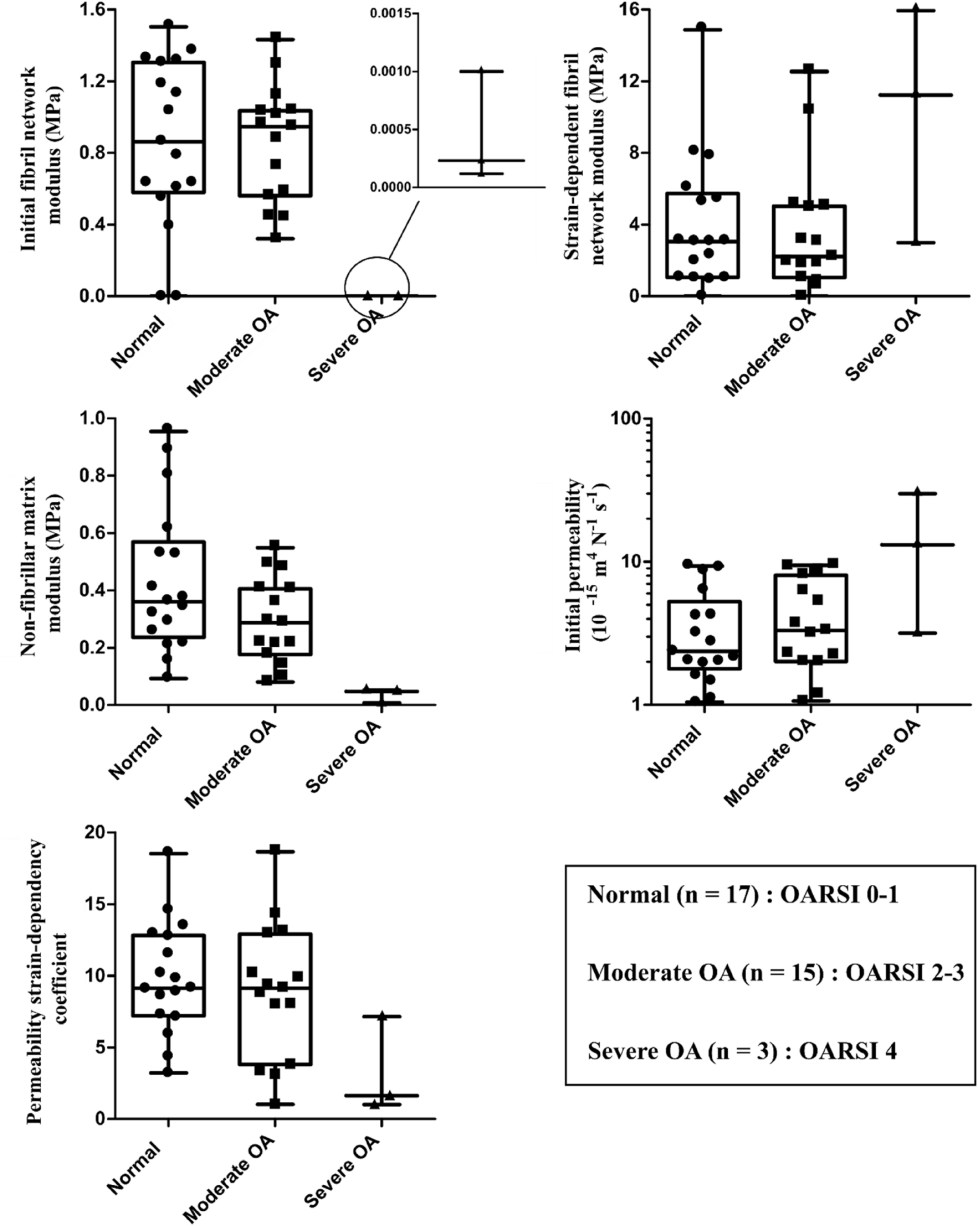
The fibril-reinforced poroelastic material parameters in the normal, moderate OA, and severe OA human femoral condyle cartilage. The statistical comparison was not possible for the severe OA group samples due to the small sample size.

The FRPE parameters were successfully fitted to the experimental stress-relaxation data (mean ± standard deviation of *R*^2^-value = 0.94 ± 0.06). We did not observe any substantial differences in the FRPE material parameters between the normal and moderately degraded cartilage samples (Figure 2). We also did not detect any essential differences in the material properties of medial and lateral compartments between normal samples and moderately degraded cartilage samples (e.g. medial normal vs. medial moderate OA, Supplementary Material Table S2).

The FRPE parameter values in the samples with severe degradation (i.e. OARSI grade 4) suggested signs of substantial cartilage deterioration, i.e., very small ${E}_{\mathrm{f}}^{0}$, small ${E}_{\mathrm{nf}}$ and $M$, and large ${k}_{0}$ were obtained compared to those in the normal or moderate OA group (and OARSI grade 0–3 groups, Figure 2). However, due to the small sample size in the OARSI grade 4 group, we were not able to statistically analyze this. The FRPE material parameters are also presented separately for each OARSI grade in the supplementary material (Supplementary Material Table S1).

### Constituent-Specific Fibril-Reinforced Poroelastic Properties in Different Joint Sites

**Figure 3:**
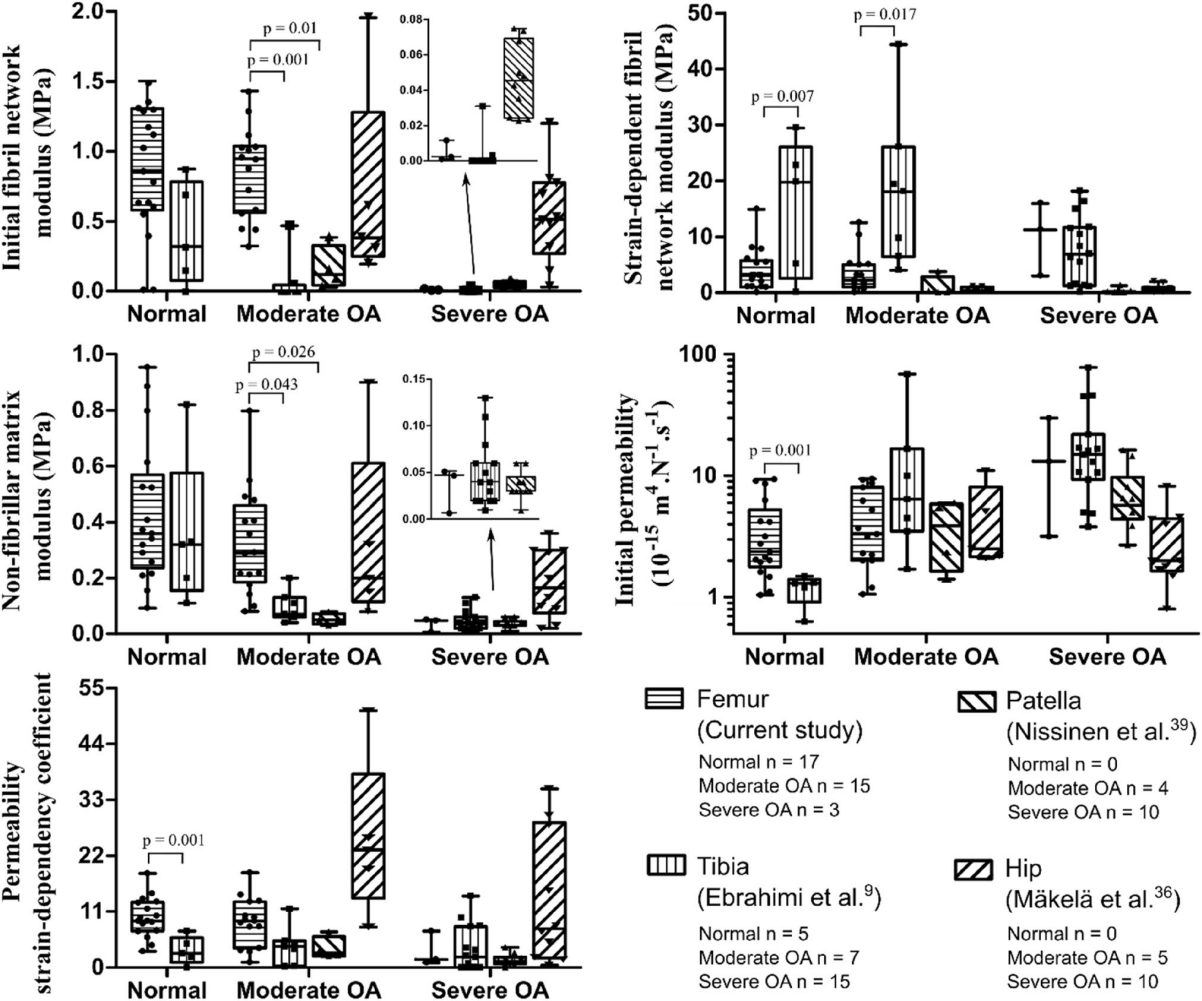
Comparison between the constituent-specific material properties of human femoral condyle cartilage with tibial, patellar and hip cartilages. The statistical comparison was not possible for the severe OA cartilage group due to the small sample size.

We also compared the femoral condyle cartilage FRPE parameters to other locations in the knee^9^,^39^ and hip^36^ joints (Figure 3). In normal femoral condyle cartilage, the initial permeability and permeability strain-dependency coefficient were 5.64 (95% CI 2.72, 8.53) ×  10^−15^ m^4^N^−1^s^−1^ and 7.71 (95% CI 3.39, 12.01) greater compared to those in the tibial plateau cartilage respectively, while the strain-dependent fibril network modulus of femoral condyle cartilage was 11.64 (95% CI 3.61, 19.67) MPa smaller compared to those in the tibial plateau cartilage (Supplementary Material Table S3).

In moderate OA, the initial fibril network modulus of the femoral condyle cartilage was 0.77 (95% CI 0.37, 1.16) and 0.68 (95% CI 0.19, 1.19) MPa greater than those of the tibial and patellar cartilage, respectively (Supplementary Material Table S3). Moreover, the non-fibrillar matrix modulus of the femoral condyle cartilage was 0.18 (95% CI 0.00, 0.36) and 0.21 (95% CI 0.02, 0.40) MPa greater than those of the tibial and patellar cartilage, respectively. In contrast, the strain-dependent fibril network modulus of the femoral condyle cartilage was smaller than that of the tibial plateau cartilage in both normal and moderate OA. For the severe OA samples, the statistical comparisons could not be conducted due to the small number of femoral condyle cartilage samples.

### Elastic and Dynamic Viscoelastic Properties

**Figure 4:**
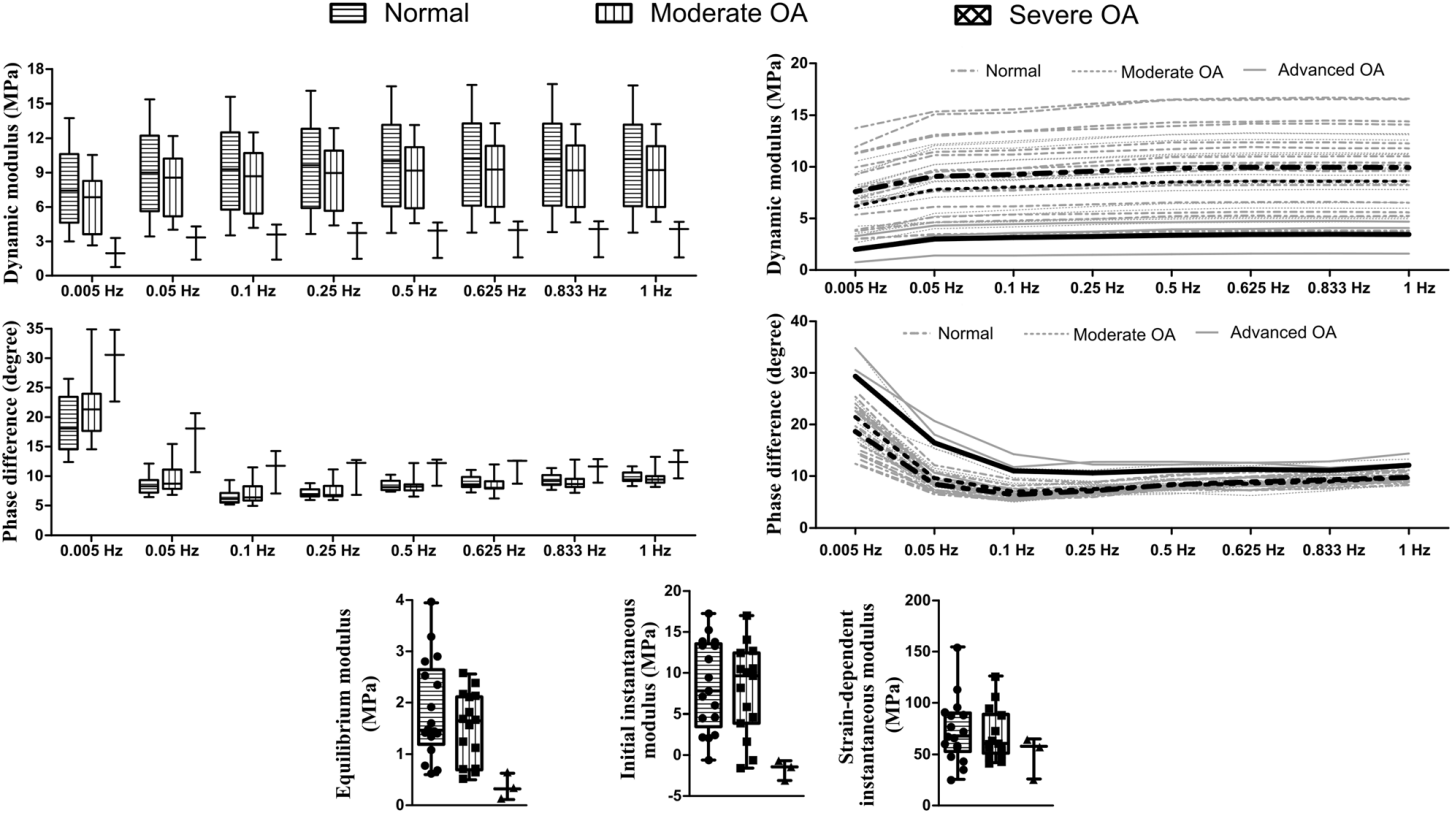
The elastic and dynamic viscoelastic material parameters of normal, moderate OA and severe OA human femoral condyle cartilage. In addition, individual trajectories (gray lines) for each sample and their corresponding means (thick lines) are shown for dynamic modulus and phase difference. The statistical comparison was not possible for the severe OA group due to the small sample size.

We did not observe any substantial differences between the normal and moderate OA groups in elastic or dynamic viscoelastic material parameters (Figure 4). These parameters were largely similar in medial and lateral compartments of normal and moderate OA samples (see Supplementary Material Tables S5, S7 and S9 for mean differences and 95% CIs).

The dynamic moduli (at all frequencies), equilibrium modulus and initial instantaneous modulus tended to be smaller, while the phase differences (at all frequencies) tended to be greater, in the severe OA compared to the normal or moderate OA group samples. However, this could not be statistically analyzed due to the small sample size in the severe OA group. The elastic (equilibrium, initial instantaneous and strain-dependent instantaneous moduli) and dynamic viscoelastic (dynamic modulus and phase difference) parameters are also presented separately for each OARSI grade at medial and lateral compartments in the supplementary material (Tables S4, S6 and S8).

## Discussion

We characterized the FRPE as well as the traditional elastic and dynamic viscoelastic properties of human femoral condyle cartilage. To our knowledge, this is the first study characterizing the constituent-specific FRPE properties for this site in the human knee. Further, these properties were also characterized at different histopathologic stages of cartilage degradation and compared to other cartilage sites reported in the literature. We hypothesized based on our earlier findings in patellar and tibial human cartilage^9^,^39^ that PG matrix- and collagen fibril network-related FRPE properties are weakened in femoral condyle cartilage at the moderate stages of OA, while the tissue permeability remains unchanged until late OA.

### Constituent-Specific Fibril-Reinforced poroelastic Properties of Normal and OA Femoral Condyle Cartilage

In contrast to our hypothesis, we observed that the FRPE material parameters were not substantially different in samples with moderate cartilage degradation compared with the samples considered normal. When examining individual trajectories of FRPE parameters (Supplementary Materials Figure S1), we observed similar values of the FRPE parameters within one knee, even if the samples from the knee were graded with different OARSI grades. This suggests a potentially important knee-specific component that influences the FRPE values more than moderate OA changes. The parameter values in severe OA cartilages, however, exhibited signs of substantial cartilage deterioration, i.e., very small ${E}_{\mathrm{f}}^{0}$, small ${E}_{\mathrm{nf}}$ and $M$, and large ${k}_{0}$ compared to those in the normal or moderate OA group. Yet, we could not statistically analyze this observation due to the small sample size in the severe OA group (most samples with severe OA were too degraded for biomechanical tests). Still, this observation (at least partially) supports our hypothesis that tissue permeability increases at the late stages of femoral condyle cartilage degeneration, although this must be verified with a larger number of samples. The possible mechanisms underlying the observed increase in permeability are: (1) loss of PGs^10^,^37^ reduces the solid-fluid interaction between the PGs and interstitial fluid,^45^ (2) loss of collagen network integrity diminishes the capacity of the tissue to trap the water,^41^ thus the interstitial fluid can flow easier.

The non-fibrillar matrix modulus, a parameter representing the stiffness of the PG matrix, was reported to be negatively associated with the OARSI grade in human tibial cartilage.^9^ Furthermore, a well-known association between the PG content and the non-fibrillar matrix modulus of cartilage was reported in literature.^9^,^23^,^25^ Interestingly, in our samples, this association was clear only in the cartilage samples with severe degradation (OARSI 4) as the non-fibrillar matrix modulus in the cartilage samples with moderate degradation (OARSI 2 and 3) did not differ from tissue considered normal (OARSI 0 and 1). This finding contradicts our previous observations in tibial cartilage^9^ which suggested that the non-fibrillar matrix modulus in moderate OA cartilage was on average 70% smaller compared to normal cartilage. This finding implies that the PG content alterations might be initiated at later stages of OA in femoral condyle cartilage compared to tibial cartilage. Previous studies suggest that the tibial cartilage experiences substantially higher strains (shear and compression) than the opposing femoral cartilage.^11^,^49^ If a similar crack is assumed to be present in both tibial and femoral cartilage, the PG loss will be intensified in tibial cartilage where deformations are higher. According to the literature, high shear strain can modulate the PG loss, especially around the lesions and cracks.^12^ This loss of PG can then cause the mechanical properties (i.e. non-fibrillar matrix) to weaken faster in tibial cartilage.

In addition, the initial fibril network modulus, a parameter suggested to represent the pretension of the collagen fibril network,^10^ exhibited no difference between normal and moderate OA tissues. This may indicate that the pretension of collagen fibril network of femoral condyle cartilage samples has not essentially been lost in moderate OA compared with normal tissue. We previously observed that the initial fibril network modulus of moderate OA human tibial cartilage was on average 83% smaller compared to normal cartilage^9^, thus our current finding contradicts our previous human tibial cartilage study. Yet, this contradiction is not surprising as we previously concluded that loss in the PG content leads to loss of collagen pretension and, in our current study, we did not observe changes in the non-fibrillar matrix modulus between healthy and moderate OA femoral cartilage. In the future, we will investigate the relationships between the structure and function to elucidate in detail how compositional and structural changes in OA are related to constituent-specific material properties.

The lack of differences between the normal and moderate OA groups may be explained by two arguments. (1) OARSI grade considers the PG network integrity while the condition of the collagen network is not evaluated. However, the mechanical tissue response in indentation geometry is strongly modulated by the superficial, parallel-oriented collagen fibrils, and this modulation is also present in the equilibrium response. Therefore, if the collagen network integrity has been the same in normal and moderate OA samples, the collagen network-related mechanical properties should also be the same. (2) The normal and moderate OA groups were unbalanced in the distribution of OARSI grades, since in the normal group most of the samples had OARSI 1 and in the moderate OA group most of the samples had OARSI 2. Samples in these groups had similar biomechanical properties.

### Constituent-Specific Fibril-Reinforced Poroelastic Properties in Different Joint Sites

We observed that the non-fibrillar matrix modulus of normal femoral condyle cartilage was not different from that of tibial cartilage, while in moderate OA femoral cartilage, it was greater compared to tibial cartilage. As this parameter is strongly associated with the PG content,^10^,^36^ the finding suggests that the OA-related changes in the PG matrix of tibial cartilage may occur before femoral condyle cartilage. Based on this finding, the PG content of human femoral condyle cartilage is likely to be higher compared to that of tibial cartilage in moderate OA.

The findings of this study showed that the initial fibril network modulus of femoral condyle cartilage appears to be greater compared to other knee joint cartilages in moderate OA, while it was not the case for normal tissue. The initial fibril network modulus has also been related to the pretension of the collagen network through swelling caused by the PG content.^10^ This study also suggests the same relationship, as we observed higher non-fibrillar matrix modulus (i.e. presumably higher PG content) and initial fibril network modulus in femoral condyle cartilage compared to tibial and patellar cartilage samples with moderate OA. In addition, we observed that, unlike in human tibial plateau cartilage, the initial collagen network modulus was not different between normal and moderate OA samples. Interestingly, several studies have also reported that (1) the collagen network in femoral condyle cartilage is mechanically superior compared to that of tibial and patellar cartilages^15^,^20^,^22^,^46^ and (2) the collagen network has a more pronounced multilaminar appearance and greater optical retardation (i.e. retardation is an indicator of fibril parallelism, higher retardation shows more parallel fibers i.e. greater degree of fibril organization) than that of tibial cartilage in human or animal samples.^2^,^21^,^32^ Combined with these literature observations, the aforementioned findings could explain why we observed (potentially) delayed alterations in the initial collagen network modulus of femoral condyle cartilage when compared to other knee joint cartilages.

The strain-dependent fibril network modulus of femoral condyle cartilage was smaller than that of tibial cartilage^9^ in both normal and moderate OA cartilage samples. This FRPE material parameter can be interpreted as how well the collagen fibrils are recruited during the application of load. As the strain-dependent fibril network modulus was smaller in femoral cartilage, our findings suggest that when we are compressing to 15% of tissue strain, we are most likely measuring a more homogenous structure (i.e. parallel collagen fibers to the surface) from the femoral condyle cartilage compared to tibial cartilage, as most of the collagen network was (presumably) intact until this depth. In addition, we observed that in the moderate OA group the initial fibril network modulus of femoral condyle cartilage was greater compared to tibial cartilage, implying that the pretension of the collagen fibrils is greater, thus, the collagen fibrils may be less crimped, and a greater portion of the collagen fibers network can contribute to tensile behavior at the initial loading phase.^9^ This results in a more linear mechanical behavior of the collagen network in femoral condyle cartilage with respect to tibial cartilage.

The results also suggest that femoral cartilage may retain its normal (arcade-like) collagen network structure in moderate OA as reflected by large initial and small strain-dependent fibril network moduli when compared to tibial (and potentially to patellar) cartilage. This could suggest that femoral cartilage may be more resistant to lesion progression compared to tibial cartilage in moderate OA, as the preservation of arcade-like collagen architecture may prevent the progression of cartilage lesions.^38^

Consistent with our findings, a 2-year clinical follow-up study indicated that cartilage erosion occurs at an earlier stage on the tibial cartilage as compared with femoral cartilage.^8^ In contrast, some clinical studies have shown that the disease deteriorates femoral cartilage more severely than the tibial cartilage during early stages of OA,^18^,^48^ which do not support our findings. However, these in-vivo clinical observations are solely based on the bulk assessment of tissue morphology and may not reflect the true mechanical, structural and compositional properties of the tissue. Moreover, different study cohorts with different gender, age and OA subtypes can make the comparison unjustified.

Inherent differences between femoral condyle and tibial cartilage structure and composition as well as adaptation to different mechanical loading environment might be the reason for our observation on different mechanical properties of femoral cartilage compared to other knee joint sites. During the tibiofemoral articulation, the tibial cartilage experiences more severe deformation and strain both in compression and in shear than the opposing femoral cartilage.^11^,^49^ Since cell metabolism and matrix synthesis are regulated by mechanical deformation,^19^,^49^ the relative differences in the deformation between articulating surfaces can be biomechanical stimulus regulating regional variations in tissue stiffness. Thus, tibial cartilage has probably been adapted to higher deformations by being less stiff. Previous studies reported higher equilibrium and dynamic moduli of femoral condyle cartilage compared to tibial cartilage in human^33^,^49^ and canine tissue.^1^ Moreover, the PG content has been reported higher in femoral condyle cartilage compared to tibial cartilage.^27^ In addition, collagen-related optical retardation (i.e. indication of collagen fibers organization) of femoral condyle cartilage was higher compared to tibial cartilage.^1^

### Elastic and Dynamic Viscoelastic Properties

We observed that the elastic and dynamic viscoelastic properties were largely similar between normal and moderate OA cartilage samples. Again, we observed a high degree of similarity within a knee, even if the samples from the two compartments were graded with different OARSI scores (Supplementary Materials Figure S2, S4 and S6). Our major advancement compared to previous studies was to include two compartments from one knee and taking this into account in the statistical analysis model where both within- and between-subject variances are modeled. We are unable to state if the similarity of the parameters within a knee is due to genetic factors or due to OA-related changes affecting the cartilage mechanical parameters of the whole knee joint, even if the histopathological grade changes, as evaluated with OARSI grades, are evident in one compartment only.

The average equilibrium modulus of human femoral condyle cartilage obtained in this study (1.57 ± 0.89 MPa) is in range of those of previous studies characterizing human femoral head cartilage (1.16 ± 0.20 MPa)^6^ or human femoral groove cartilage (1.24 ± 0.47 MPa).^26^ The average dynamic modulus measured at 1 Hz in this study (8.94 ± 3.85 MPa) is consistent with that of the most recent study performed on human femoral condyle cartilage at the same frequency (7.71 ± 4.62 MPa).^28^

The dynamic moduli were not substantially different between normal samples and samples with moderate OA signs, while a few highly degenerated (OARSI grade 4) samples had considerably lower dynamic moduli. The collagen fibril network and the tissue permeability are important contributors to the dynamic properties.^34^,^42^,^43^ The initial fibril network modulus in severe OA samples was negligible (0.00001 ± 0.00001 MPa). This was also seen in the initial instantaneous modulus (the intercept of the fitted line) of the severe OA group. These findings suggest that the pretension in the collagen network of severely degraded OA cartilage is completely lost, presumably due to the loss of superficial and middle zone (PG content and/or collagen fibers), ultimately affecting the function of collagen fibrils to maintain fluid pressurization and carry loads effectively.

Interestingly, we observed no substantial differences in the phase difference (tissue viscosity) between normal and moderate OA, while the phase difference of the severe OA group seemed to be much higher. The lack of changes in the phase difference of normal and moderate OA at different frequencies indicates that cartilage is still capable to trap interstitial fluid and pressurize. This further supports our earlier deduction that the collagen fibril network^10^,^45^ remained presumably relatively intact, especially during moderate OA. This deduction was already supported by our other findings in this study; we observed no substantial changes in the initial and strain-dependent fibril network moduli or the initial and strain-dependent instantaneous moduli between normal and moderate OA, strongly suggesting a nearly intact collagen network during earlier stages of OA in femoral condyle cartilage. Further, the disorganization and degradation of the collagen fibril network have been suggested to increase tissue permeability (which can be observed as reduced fluid pressurization in tissue).^42^,^45^ In the current study, the permeability of cartilage was not essentially different between normal and moderate OA as graded by histopathology, supporting the observation of phase difference. This further suggests that the collagen fibers remained intact in our moderate OA cartilage samples. However, this speculation must ultimately be assessed using compositional and structural measurements (e.g. microscopy and spectroscopy) and this will be done in our future study.

## Limitations

The main weakness of our study is the low number of severely degenerated OARSI grade 4 samples and moderately degenerated OARSI 3 samples. Therefore, we were able to conduct statistical analyses only between the normal and moderate OA pooled groups. Moreover, the moderate OA group included only three samples graded as OARSI 3, thus it was more representative of healthier OARSI grade 2 samples. We acknowledge that having those two groups with unevenly distributed OARSI grades may have affected the results.

We also acknowledge that the samples that are harvested from different donors with different gender, age and OA subtypes might affect the validity of the comparison of mechanical properties between human femoral cartilage (this study) and other cartilage sites,^9^,^39^ and thus, the conclusion made based on these comparisons may not be necessarily generalized.

### Future Considerations

The findings of this study help in designing better tissue-engineered constructs, in which investigators seek to fabricate tissue-engineered constructs that could mimic the behavior of native tissue. Here, we had a relatively large group of normal samples (specifically OARSI grade 1) obtained from young subjects in some cases and this study elucidated the mechanical properties of each constituent for those samples. However, care should be taken when applying our findings to *in vivo* tissue engineering efforts since the properties of implanted tissue-engineered materials (and surrounding tissue) might be affected when the body invades them (e.g. scar tissue remodeling, interface adhesion). Moreover, the condition of the surrounding tissue must be taken into account to avoid abnormal stress-strain distributions in tissue-construct interfaces.

Finally, despite the sophistication of recent computational knee joint models,^13^ they typically use material properties obtained from animals. The findings of this study showed differences in cartilage mechanical properties of different sites in the knee joint. By implementing the true cartilage properties in the knee computational model, the local areas susceptible to OA can be more precisely detected. Moreover, one could employ a computational multi-scale modeling approachyy^44^ combined with the findings of this study to predict cell deformations in each cartilage site. Therefore, the knowledge established in this study can naturally improve the accuracy of knee joint models to predict susceptible areas to OA development.

## Conclusion

The present study provides novel information about the constituent-specific material properties of human femoral condyle cartilage, how they compare with other cartilage sites, and how they are altered at different stages of OA. The results suggest that alterations in the femoral condyle constituent-specific mechanical material properties are not evident in moderate stage OA, while they appear to be substantially altered in late OA. The mechanical properties related to non-fibrillar and fibrillar matrices of femoral condyle cartilage seem to be partially different from that of other knee joint cartilages in moderate OA, possibly leading to a delay in deterioration of femoral condyle cartilage properties as compared to other knee joint cartilages.

## Supplementary Information

Below is the link to the electronic supplementary material.

Supplementary file1 (PDF 1504 kb)
