# Supplementary material for: Elastic, Dynamic Viscoelastic and Model-Derived Fibril-Reinforced Poroelastic Mechanical Properties of Normal and Osteoarthritic Human Femoral Condyle Cartilage
Source: Ann Biomed Eng. 2021 Aug 2;49(9):2622–34. doi: 10.1007/s10439-021-02838-4 (PMC8455392; doi:10.1007/s10439-021-02838-4)
Supplement: Supplementary file 1 — Supplementary file1 (PDF 1504 kb) [file 10439_2021_2838_MOESM1_ESM.pdf]

Supplementary materials for:

# Elastic, dynamic viscoelastic and model-derived fibril-reinforced poroelastic mechanical properties of normal and osteoarthritic human femoral condyle cartilage

Mohammadhossein Ebrahimi[1,2]†, Mikko Finnilä[2], Aleksandra Turkiewicz[3], Martin Englund[3], Simo Saarakkala[2], Rami K. Korhonen[1], Petri Tanska[1]

[1]Department of Applied Physics, University of Eastern Finland, Kuopio, Finland; [2]Research Unit of Medical Imaging, Physics and Technology, Faculty of Medicine, University of Oulu, Oulu, Finland; [3]Faculty of Medicine, Department of Clinical Sciences Lund, Orthopaedics, Clinical Epidemiology Unit, Lund University, Lund, Sweden

**†Corresponding author:**
Mohammadhossein Ebrahimi
Department of Applied Physics
University of Eastern Finland
POB 1627, FI-70211 Kuopio
Finland
Tel. +358-449691559
E-mail: mohammadhossein.ebrahimi@uef.fi , ebrahimi.bioengineering@gmail.com

# Materials and methods

## Details on sample preparation

Thirty-five osteochondral samples from medial and lateral femoral condyle of total knee replacement (TKR) patients due to medial compartment tibiofemoral OA (number of samples = 16, number of subjects = 14, age 64.5 ± 7.9 years, age range 53–79 years, 6 men and 8 women) and healthy donors without known clinical knee OA or rheumatoid arthritis (number of samples = 19, number of subjects = 10, age 53.4 ± 18.8 years, age range 18–77 years, 5 men and 5 women) were obtained by a hole saw (diameter = 4 mm). Initially, our sample set consisted of 50 samples but most of the medial condyle samples from TKR patients and one sample from healthy donors were not measurable in biomechanical indentation geometry due to full-thickness cartilage loss, thus they were not included in the study.

Surgeries were conducted in Trelleborg Hospital, Trelleborg, Sweden while donor samples were obtained from Skåne University Hospital, Lund, Sweden. All samples were obtained within 48 h postmortem and frozen at -80 °C within 2 hours of extraction in phosphate buffered saline (PBS). Afterwards, the samples were transferred from Sweden to Finland, while kept frozen in dry ice (~-78 $^0$C). They were then kept at -23 °C until conducting experiments. The storage of samples at this temperature should not cause changes in composition[25,28] or mechanical properties of the tissue[27].

## Details on mechanical indentation testing

The osteochondral sample in PBS was thawed at room temperature for 15 minutes before starting the measurements. Based on literature[27], this was considered enough to thaw the sample. Cartilage thickness from each osteochondral sample was measured from the surface to the cartilage-bone interface using an optical stereomicroscope (9 1.6, Zeiss, STEMI, SV8, Germany). The measurements were conducted from 4 different quarters on the sample side. The mean value was then calculated to represent the sample thickness. We then flattened the bone surface using sandpaper (Mirox P80, Mirka Oy, Uusikaarlepyy, Finland) to reduce roughness and increase adherence of bone to the measurement chamber. The bone end was then glued (Loctite Precision, Henkel AG, Düsseldorf, Germany) on the metallic bottom of a custom-made transparent acrylic chamber, which was filled with PBS.

A custom-made high-precision linear servo-motorized indentation device (Newport PM500-C Precision Motion Controller, Newport PM1A1798 Actuator, Irvine, CA, USA) equipped with a 1000 g load cell (Honeywell afterwaModel 31/AL311BL, Columbus, OH, USA) and a custom-made cylindrical plane-ended metal indenter (diameter = 0.73 mm) were used to measure the mechanical response of the samples. This indenter diameter is small enough to minimize effects from the sample edges[26].

Prior to starting the measurement, a pre-stress of 12.5 kPa was applied to establish a proper sample-indenter contact for consistent and repeatable measurements, similarly as in previous studies[13,19]. We allowed the sample to equilibrate for about 15 minutes[3,9] and checked that pre-stress remained at 12.5 kPa. We then set the zero-strain level (reference strain). Subsequently, a 4-step stress-relaxation protocol was applied. Each step included the application of a 5% compressive strain (of the remaining cartilage thickness) followed by 15 minutes of relaxation[19]. In preliminary tests, we observed that this relaxation time is enough to reach equilibrium. We applied strain rate of 100%/s to ensure maximum fluid pressurization (and collagen fibers involvement) in the tissue. The same strain rate has also been used in previous studies[19,21]. A recent review article suggested that testing at higher strain rates is dominated by initial fluid pressurization, whereas slower strain rates (e.g., 0.1%/s) allow for some energy dissipation by fluid flow[24]. Moreover, strain-rates of this magnitude are known to occur during daily activities[2]. The multi-step protocol was chosen to investigate the nonlinear properties from the peak forces (i.e. nonlinear fibril network and instantaneous modulus) and relaxation phases (i.e. nonlinear strain-dependent permeability) as well as the linear stress-strain response from the equilibrium points. Some in-vivo studies have reported nominal cartilage strain of up to 20% during gait cycle[1,18,22]. Moreover, previous studies have suggested that ~20% total strain does not cause plastic deformation or damage to cartilage[13,19]. After the stress-relaxation measurement (the sample at the final strain of the stress-relaxation experiment), a dynamic sinusoidal test was conducted using a 2% strain amplitude (of the remaining thickness) with frequencies of 0.005, 0.05, 0.1, 0.25, 0.5, 0.625, 0.833 and 1 Hz (four cycles for each frequency). Based on earlier studies[16,29], this frequency regime was assumed to demonstrate frequency-dependent changes in the dynamic modulus and phase difference. The 2% strain level provided us with a measurable response in the cartilage tissue, while keeping the total strain level within the safe region[13,19].

## Details on elastic and dynamic viscoelastic properties

The equilibrium modulus of cartilage was calculated from a slope of a linear least-squares fit to the stress-strain points at the equilibrium (Figure 1, middle column). The modulus was corrected using the Hayes correction factor[6] as follows:

$$E = \frac{(1 - \nu^2)\pi a}{2\kappa h} F, \quad (1)$$

where $F$ is the indenter force, E is the Young's modulus, ν is the Poisson's ratio, a is the indenter radius, h is the sample thickness and κ is a non-dimensional constant (determined based on the Poisson's ratio and the aspect ratio (i.e. a/h)). We set Poisson's ratio at equilibrium to 0.3[11]. To obtain the dynamic moduli at different frequencies, the stress and strain amplitudes were calculated from each cycle and averaged over the 4 measured consecutive cycles. We also corrected the dynamic moduli using the Hayes correction factor[6]. Here, we assumed the sample to be incompressible during the dynamic loading (as fluid is pressurized due to high loading rate), thus Poisson's ratio was set to 0.5[10]. The Fourier transform was used to extract the frequency content of dynamic data. The phase angle was extracted at a frequency at which the power amplitude peaks. The phase difference was calculated by subtracting the displacement and force phase angles. The instantaneous modulus was calculated from the peak stress values at each step (Figure 1, middle column). Thus, we obtained data points for the instantaneous modulus as a function of strain. Accordingly, a linear least-squares line was fitted to the data points of instantaneous modulus vs. applied strain and used to determine the initial instantaneous modulus (intercept) and strain-dependent instantaneous modulus (slope). These moduli were also corrected using the Hayes correction factor[6] with Poisson's ratio set to 0.5[10] as the sample was assumed to be incompressible due to the high loading rate.

## Details on finite element analysis and optimization

### *Material model*

Cartilage tissue was modeled using the FRPE material model, in which articular cartilage is composed of an elastic fibrillar matrix (representing the collagen fiber network) and a porous hyperelastic non-fibrillar matrix (representing the PG matrix), filled with fluid.

The collagen network was modeled with 4 organized collagen fibrils (primary fibrils) and 13 randomly oriented fibrils (secondary fibrils)[19,32]. The density ratio of the primary fibrils to the secondary fibrils was fixed to 12.16[8,19]. The compressive stress-strain behavior of the fibrils was set to zero, while in tension the following non-linear stress-strain behavior was assumed:

$$\sigma_{\mathrm{f}} = \frac{1}{2} E_{\mathrm{f}}^{\varepsilon} {\varepsilon_{\mathrm{f}}}^{2} + E_{\mathrm{f}}^{0} \varepsilon_{\mathrm{f}}, \tag{2}$$

where $\sigma_{\mathrm{f}}$ and $\varepsilon_{\mathrm{f}}$ are stress and strain of the fibril, $E_{\mathrm{f}}^{0}$ is the initial fibril network modulus and $E_{\mathrm{f}}^{\varepsilon}$ is the strain-dependent fibril network modulus[17]. Furthermore, the non-fibrillar matrix was modeled using the Neo-Hookean hyperelastic material model. The below formulation for the non-fibrillar matrix stress was used:

$$\boldsymbol{\sigma}_{\mathrm{nf}} = \frac{1}{2} K_{\mathrm{nf}} (\boldsymbol{J} - \boldsymbol{J}^{-1}) \mathbf{I} + \frac{G_{\mathrm{nf}}}{J} \left( \mathbf{F}\mathbf{F}^{\mathrm{T}} - J^{\frac{2}{3}} \mathbf{I} \right), \tag{3}$$

where $\boldsymbol{\sigma}_{\mathrm{nf}}$ is the stress tensor of the non-fibrillar matrix, $G_{\mathrm{nf}}$ and $K_{\mathrm{nf}}$ are the shear and bulk moduli of the non-fibrillar matrix, respectively, **F** is the deformation gradient tensor, *J* is the determinant of the **F** and **I** is the unit tensor[31]. The bulk and shear moduli of the non-fibrillar matrix can be formulated based on Young's modulus ($E_{\mathrm{nf}}$) and Poisson's ratio ($\nu_{\mathrm{nf}} = 0.42$, based on[12,19]) of the non-fibrillar matrix:

$$K_{\mathrm{nf}} = \frac{E_{\mathrm{nf}}}{3(1 - 2\nu_{\mathrm{nf}})}, \tag{4}$$

$$G_{\mathrm{nf}} = \frac{E_{\mathrm{nf}}}{2(1 + \nu_{\mathrm{nf}})}, \tag{5}$$

Regarding the fluid flow behavior, Darcy's law[7] was recruited to describe the fluid flow inside the porous matrix as follows:

$$\boldsymbol{q} = -k \nabla p, \tag{6}$$

where $\boldsymbol{q}$ is the fluid flow flux, $k$ is the (hydraulic) permeability of the material and $\nabla p$ is the (fluid) pressure gradient. Darcy's law is valid with laminar and low velocity flows, which is true in most biological tissues[5]. The deformation in the porous material causes a change in the void ratio (the proportion of the fluid volume to the solid volume), and consequently, changes in the permeability[30], which is formulated in the following way:

$$k = k_0 \left(\frac{1+e}{1+e_0}\right)^M , \tag{7}$$

where $k$ and $k_0$ are the current and initial values for the permeability, and $e$ and $e_0$ are the current and initial values for the void ratio, respectively. $M$ is a constant describing the void-ratio (or deformation) -dependency of permeability[5,30,32]. The initial void ratio $e_0$ which is the ratio of the fluid volume to the solid volume was set to 3, based on literature[14].

*Model construction and boundary conditions*

Sample-specific axisymmetric models were built in Abaqus (V6.14, Dassault Systèmes Simulia Corp., Providence, RI). The samples were meshed using linear axisymmetric pore pressure continuum elements (element type CAX4P). A mesh convergence test was conducted to ensure a proper mesh size. The structure and composition were assumed homogenous (i.e. collagen fiber network orientation parallel to the cartilage surface, homogeneous PG and collagen contents, and constant void ratio) to obtain material properties independent from the composition and structure of the tissue, similarly as done before[4,12,20]. The compression of the indenter was simulated by a displacement boundary condition on the cartilage surface for computational efficacy. The contact between the lateral edge of the indenter (an analytical rigid surface) and cartilage surface was modeled using a frictionless hard contact (both in normal and tangential direction; during contact the separation of surfaces was allowed only in tangential directions) to prevent folding of the cartilage mesh. Free draining was allowed from non-contacting surfaces (i.e. pore pressure = 0), while cartilage-indenter contact was modeled impermeable. The axisymmetric boundary condition was applied on the symmetry axis of the sample, *i.e.*, lateral displacement of the nodes at the symmetry axis was fixed and fluid was not allowed to flow through the symmetry axis[19]. Axial and lateral displacements of the bottom nodes of cartilage were fixed because the subchondral bone was considered rigid. The fluid flow was restricted through the cartilage-bone interface. The models were solved using the soils consolidation analysis in Abaqus.

The following material parameters of the FRPE model ($E_\mathrm{f}^0, E_\mathrm{f}^\varepsilon, E_\mathrm{nf}, k_0, M$) were obtained by fitting the force-time response of the second and third steps of the model to the corresponding steps of the experimental stress-relaxation test. The first stress-

relaxation step was simulated in the model but not included in the fitting procedure as this step was considered not to be always in a proper contact[19]. In addition, if more than two steps are used in an optimization procedure, the high level of nonlinearities resulting from inherent inhomogeneities of cartilage may become dominant, thus leading to a poor fit quality with the current material model. The data fitting was conducted using the Nelder-Mead simplex algorithm (*fminsearch*) implemented in Matlab v7.10.0 (The MathWorks, Inc., Natick, MA)[15]. The objective function for the optimization routine was based on the normalized mean squared error between the simulated and experimental data. This function was modified with a weighting term that improves the fitting of the peak force responses (the value for the weighting factor *w* = 1 was selected based on preliminary simulations), thus the used objective function was:

$$\delta\bar{F} = \frac{1}{n}\sum_{i=1}^{n}\left(\frac{F_i^{\mathrm{sim}} - F_i^{\mathrm{exp}}}{F_i^{\mathrm{exp}}}\right)^2 + w\frac{1}{m}\sum_{j=1}^{m}\left(\frac{F_{j,\mathrm{p}}^{\mathrm{sim}} - F_{j,\mathrm{p}}^{\mathrm{exp}}}{F_{j,\mathrm{p}}^{\mathrm{exp}}}\right)^2, \qquad (8)$$

where $F_i^{\mathrm{sim}}$ and $F_i^{\mathrm{exp}}$ are simulated and experimental force values, $F_{j,\mathrm{p}}^{\mathrm{sim}}$ and $F_{j,\mathrm{p}}^{\mathrm{exp}}$ are peak force values obtained from simulation and experiment, and $n$ and $m$ correspond to the total number of data points and the number of peak data points ($m = 2$ in our case), respectively.

**Details on statistical analyses**

We analyzed the dependent variables (*i.e.* the FRPE material parameters ($E_{\mathrm{f}}^{0}, E_{\mathrm{f}}^{\varepsilon}, E_{\mathrm{nf}}, k_0, M$) as well as elastic and dynamic viscoelastic material properties) to compare mean values between the groups. A linear mixed-effects (LME) model was used. This statistical model accounts for the dependency of the samples obtained from the same subject (donor or patient). Subjects in each group were set as a mixed (random) effect while the OA progression group (i.e. normal or moderate OA), the compartment (i.e. medial or lateral) and the interaction of the variables were set as fixed variables. The LME models were age and BMI adjusted for group-wise comparisons of femoral condyle cartilage samples.

Regarding the dynamic moduli measured as a function of frequency, we analyzed it using linear splines, because of the non-linear association between frequency and the outcomes (dynamic moduli). The LME model with dynamic moduli as outcomes, and

the three linear splines (with knots at 0.05 Hz and 0.5), compartment (medial or lateral) and OA status (moderate OA or normal) as independent variables was used. We included the subject and compartment as random effects and allowed for random slopes for the first spline (as there was no relevant variability in the slopes of the other two splines, see supplementary Figure S3). We assumed an unstructured covariance matrix and used Satterwhite's method for the calculation of degrees of freedom.

For the analysis of phase differences, we treated the frequency as a categorical variable with frequencies of 0.25 Hz and higher as one group, based on inspection of individual trajectories (supplementary Figure S5). We fitted a linear mixed model with compartment, group, frequency, and their interactions as independent variables. We included random intercepts for the subject, compartment and random slopes. We allowed for heteroskedastic errors as the variability was higher at a frequency of 0.005 Hz. We assumed an unstructured covariance matrix and used Satterwhite's method for the calculation of degrees of freedom. The analyses were repeated with additional adjustments for age and BMI.

Moreover, an age-adjusted LME model was used to compare the FRPE properties of femoral condyle samples to those of other human cartilages (i.e. femoral condyle vs. tibial, patellar and hip joint cartilage). Subjects in each group were included as a random effect while the sites (i.e. femoral (current study), tibial[4] (samples = 27, subjects = 7), patellar[23] (samples = 14, subjects = 6), and hip joint cartilage[20] (samples = 15, subjects = 8)) and the OA status (i.e., normal and moderate OA) were set as fixed variables. The patients from which the samples are harvested in each of the abovementioned literature references are different from the current study.

## Results

### Detailed analyses of fibril-reinforced poroelastic (FRPE) parameters

Descriptive data on FRPE material parameters for each group is shown in Figure S1 (each color represents samples from the same knee, except red color, which represents subjects contributing with only one sample). Moreover, descriptive data on FRPE material parameters for each OARSI grade are shown in Table S1.

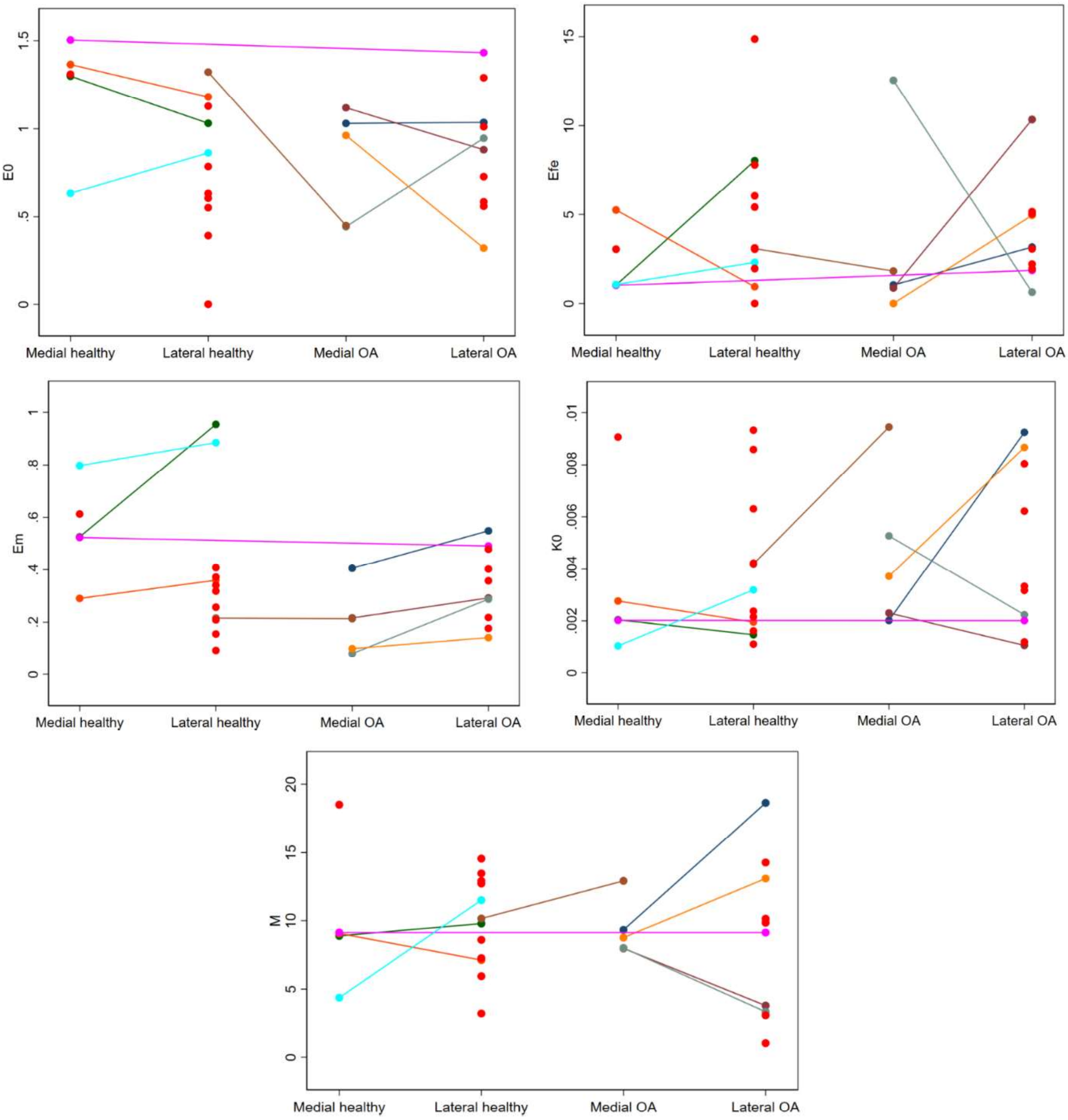


Figure S1: Descriptive data of FRPE parameters grouped according to health status in different locations (medial vs. lateral). Each color represents samples from the same knee, except red color, which represents subjects contributing with only one sample.

Table S1: Descriptive data on the FRPE material properties of human femoral condyle cartilage. The numbers are mean (SD) for medial, lateral and both compartments.

| FRPE parameter | OARSI 0 (*n* = 5) | OARSI 1 (*n* = 12) | OARSI 2 (*n* = 12) | OARSI 3 (*n* = 3) | OARSI 4 (*n* = 3) |
|---|---|---|---|---|---|
| $E_f^0$ (MPa) | 1.05 ± 0.26 | 0.78 ± 0.52 | 0.79 ± 0.32 | 1.09 ± 0.30 | 0.00001 ± 0.00001 |
| Med | 1.30 | 1.20 ± 0.39 | 0.80 ± 0.33 | - | 0.00001 ± 0.00001 |
| Lat | 0.99 ± 0.25 | 0.56 ± 0.44 | 0.79 ± 0.34 | 1.09 ± 0.30 | - |
| $E_f^\varepsilon$ (MPa) | 4.98 ± 6.40 | 3.60 ± 2.29 | 3.49 ± 3.32 | 4.28 ± 5.28 | 10.06 ± 6.55 |

| | | | | | |
|---|---|---|---|---|---|
| Med | 1.04 | 2.60 ± 2.00 | 3.26 ± 5.23 | - | 10.06 ± 6.55 |
| Lat | 5.96 ± 6.93 | 4.10 ± 2.07 | 3.65 ± 1.38 | 4.28 ± 5.28 | - |
| $E_{\mathrm{nf}}$ (MPa) | 0.45 ± 0.31 | 0.42 ± 0.24 | 0.28 ± 0.15 | 0.36 ± 0.12 | 0.04 ± 0.02 |
| Med | 0.52 | 0.55 ± 0.21 | 0.20 ± 0.13 | - | 0.04 ± 0.02 |
| Lat | 0.43 ± 0.36 | 0.35 ± 0.24 | 0.33 ± 0.16 | 0.36 ± 0.12 | - |
| $k_0 \times 10^{-15}$ ($m^4N^{-1}s^{-1}$) | 3.29 ± 2.98 | 3.92 ± 2.89 | 5.22 ± 2.02 | 1.77 ± 0.62 | 15.43 ± 13.56 |
| Med | 2.04 | 3.72 ± 3.63 | 4.55 ± 3.03 | - | 15.43 ± 13.56 |
| Lat | 3.59 ± 3.35 | 4.01 ± 2.73 | 5.69 ± 3.15 | 1.77 ± 0.62 | - |
| $M(-)$ | 9.46 ± 2.16 | 9.99 ± 4.45 | 9.76 ± 4.75 | 5.43 ± 3.21 | 3.25 ± 3.28 |
| Med | 8.88 | 10.26 ± 5.92 | 9.39 ± 2.04 | - | 03.25 ± 3.28 |
| Lat | 9.60 ± 2.46 | 9.85 ± 4.00 | 10.02 ± 6.20 | 5.43 ± 3.21 | - |

Note that when medial and lateral compartments are pooled, the “standard deviation” is a combination of within and between subject variability.

The results of linear mixed model analyses comparing FRPE parameters between normal and moderate OA groups at medial and lateral femoral condyle cartilage are shown in Table S2.

The differences (with 95%CIs) in fibril-reinforced poro(visco-)elastic properties of femoral condyle cartilage and other sites (tibial, patellar and hip joint cartilage) in different OA stages can be seen in Table S3.

Table S2: The differences (with 95%CIs) in the fibril-reinforced poroelastic properties between the normal and moderate OA groups at medial and lateral femoral condyle cartilage.

| | Parameter | Crude | Adjusted for age and BMI |
|---|---|---|---|
| Moderate OA vs normal, overall | $E_f^0$ | -0.13 (-0.42, 0.17) | -0.18 (-0.51, 0.14) |
| | $E_f^{\varepsilon}$ | 0.04 (-2.85, 2.93) | 0.19 (-3.17, 3.54) |
| | $E_{\mathrm{nf}}$ | -0.08 (-0.23, 0.07) | -0.03 (-0.18, 0.12) |
| | $k_0$ | 0.00 (0.00, 0.00) | 0.00 (0.00, 0.00) |
| | $M$ | -0.88 (-4.40, 2.63) | -1.21 (-5.30, 2.88) |
| Moderate OA vs normal, medial compartment | $E_f^0$ | -0.42 (-0.91, 0.07) | -0.48 (-0.93, 0.03) |
| | $E_f^{\varepsilon}$ | 0.97 (-3.77, 5.71) | 1.15 (-4.06, 6.36) |
| | $E_{\mathrm{nf}}$ | -0.12 (-0.34, 0.10) | -0.09 (-0.32, 0.13) |
| | $k_0$ | 0.00 (0.00, 0.00) | 0.00 (0.00, 0.00) |
| | $M$ | -0.64 (-6.38, 5.10) | -1.05 (-7.31, 5.21) |
| Moderate OA vs normal, lateral compartment | $E_f^0$ | 0.17 (-0.16, 0.50) | 0.11 (-0.23, 0.46) |
| | $E_f^{\varepsilon}$ | -0.88 (-4.09, 2.33) | -0.78 (-4.35, 2.80) |
| | $E_{\mathrm{nf}}$ | -0.05 (-0.21, 0.12) | 0.03 (-0.13, 0.20) |

| | | | |
|---|---|---|---|
| | $k_0$ | 0.00 (0.00, 0.00) | 0.00 (0.00, 0.00) |
| | $M$ | -1.13 (-5.01, 2.76) | -1.37 (-5.70, 2.97) |

$E_f^0$: initial fibril network modulus, $E_f^\varepsilon$: strain-dependent fibril network modulus, $E_{nf}$: non-fibrillar matrix modulus, $k_0$: initial permeability, $M$: permeability strain-dependency coefficient

Table S3: The differences (with 95%CIs) in the fibril-reinforced poroelastic properties of femoral condyle cartilage and other sites in different OA stages.

| Parameter | Status | Site compared | Adjusted for age |
|---|---|---|---|
| Initial fibril network modulus $E_f^0$ | Normal | Tibia | 0.45 (-0.11, 1.00) |
| | Moderate OA | Tibia | 0.77 (0.37, 1.16) |
| | | Patella | 0.68 (0.19, 1.19) |
| | | Hip | 0.19 (-0.11, 0.50) |
| Strain-dependent fibril network modulus $E_f^\varepsilon$ | Normal | Tibia | -11.64 (-19.67, -3.61) |
| | Moderate OA | Tibia | -15.72 (-28.69, -2.75) |
| | | Patella | -2.32 (-16.15, 11.50) |
| | | Hip | 3.48 (-1.45, 8.41) |
| Non-fibrillar matrix modulus $E_{nf}$ | Normal | Tibia | 0.09 (-0.21, 0.39) |
| | Moderate OA | Tibia | 0.18 (0.00, 0.36) |
| | | Patella | 0.21 (0.02, 0.40) |
| | | Hip | 0.34 (-0.10, 0.17) |
| Initial permeability $k_0$ | Normal | Tibia | 5.64 (2.72, 8.53) |
| | Moderate OA | Tibia | -11.63 (-27.21, 3.95) |
| | | Patella | 5.68 (-18.21, 19.34) |
| | | Hip | 0.40 (-8.01, 8.88) |
| Permeability strain-dependency coefficient $M$ | Normal | Tibia | 7.71 (3.39, 12.01) |
| | Moderate OA | Tibia | 4.14 (-1.05, 9.33) |
| | | Patella | 4.72 (-1.53, 10.97) |
| | | Hip | -7.54 (-14.97, -0.12) |

## Detailed analyses of equilibrium, initial and strain-dependent instantaneous moduli

Descriptive data on equilibrium, initial and strain-dependent instantaneous moduli for each group shown in figure S2 (each color represents samples from the same knee, except red color, which represents those subject contributing with only one sample). Moreover, descriptive data on FRPE material parameters for each OARSI grade are shown in Table S4.

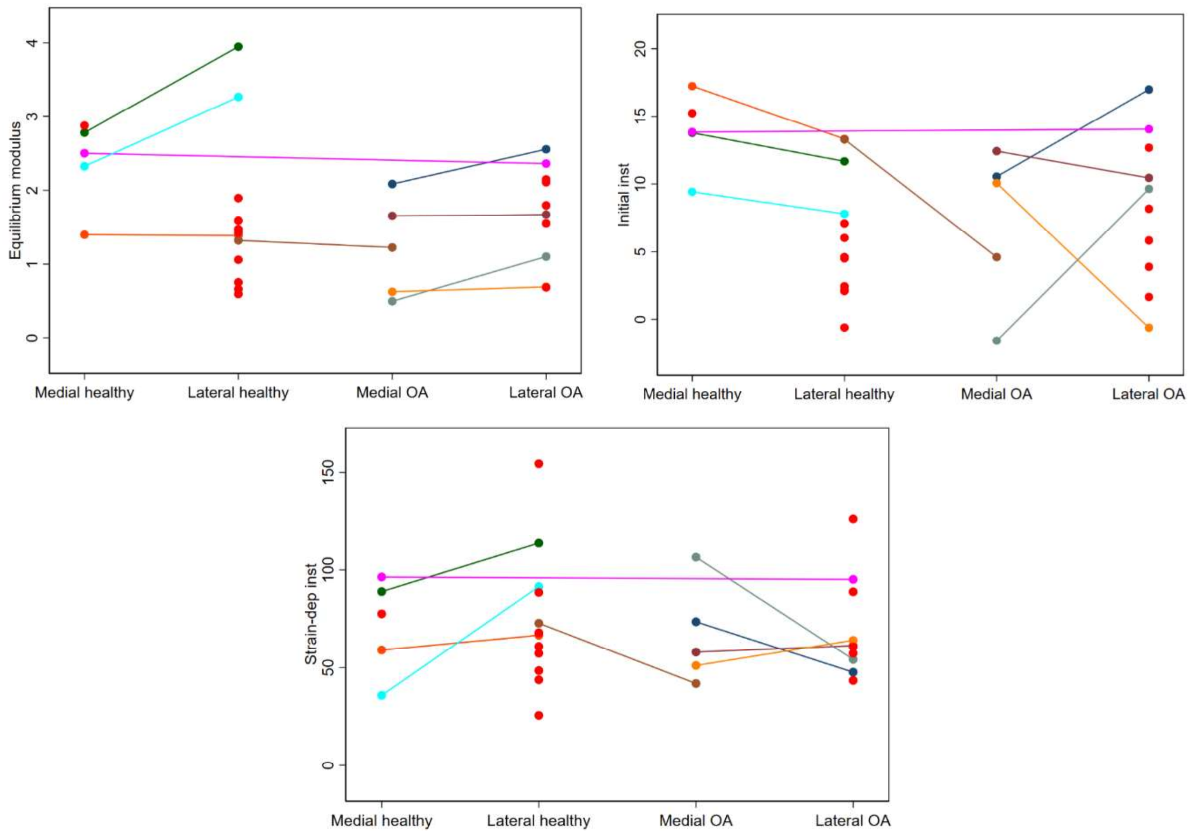

Figure S2: Equilibrium, initial and strain-dependent instantaneous moduli grouped according to health status (normal and moderate OA) in different locations (medial vs. lateral)

Table S4: The elastic material properties as well as sample thickness of human femoral condyle cartilage. The numbers are mean (SD) for medial, lateral and both compartments.

| Parameter | OARSI 0 (*n* = 5) | OARSI 1 (*n* = 12) | OARSI 2 (*n* = 12) | OARSI 3 (*n* = 3) | OARSI 4 (*n* = 3) |
|---|---|---|---|---|---|
| $E_{\mathrm{eq}}$ (MPa) | 2.05 ± 1.31 | 1.75 ± 0.84 | 1.47 ± 0.71 | 1.71 ± 0.63 | 0.35 ± 0.26 |
| Med | 2.78 | 2.27 ± 0.63 | 1.21 ± 0.67 | - | 0.35 ± 0.26 |
| Lat | 1.87 ± 1.43 | 1.48 ± 0.83 | 1.64 ± 0.72 | 1.71 ± 0.63 | - |
| $E_{\mathrm{inst}}^{0}$ (MPa) | 9.39 ± 5.08 | 8.08 ± 5.77 | 7.05 ± 5.74 | 11.38 ± 2.35 | -1.74 ± 1.23 |
| Med | 13.78 | 13.93 ± 3.31 | 7.21 ± 5.71 | - | -1.74 ± 1.23 |
| Lat | 8.29 ± 5.14 | 5.15 ± 4.27 | 6.93 ± 6.20 | 11.38 ± 2.35 | - |
| $E_{\mathrm{inst}}^{\varepsilon}$ (MPa) | 98.32 ± 36.77 | 63.10 ± 22.68 | 68.23 ± 26.31 | 70.17 ± 21.89 | 49.63 ± 20.80 |
| Med | 88.91 | 67.16 ± 25.90 | 66.15 ± 25.34 | - | 49.63 ± 20.80 |
| Lat | 100.67 ± 42.02 | 61.06 ± 22.50 | 69.71 ± 28.89 | 70.17 ± 21.89 | - |
| Thickness (mm) | 2.72 ± 0.87 | 2.60 ± 0.50 | 2.42 ± 0.51 | 2.61 ± 1.04 | 1.75 ± 0.47 |
| Med | 3.19 | 2.30 ± 0.42 | 2.03 ± 0.47 | - | 1.75 ± 0.47 |
| Lat | 2.61 ± 0.96 | 2.75 ± 0.49 | 2.69 ± 0.34 | 2.61 ± 1.04 | - |

Note that when medial and lateral compartments are pooled, the “standard deviation” is a combination of within and between subject variability.

The results of linear mixed model analyses comparing elastic parameters between normal and moderate OA groups at medial and lateral femoral condyle cartilage are shown in Table S5.

Table S5: The differences (with 95%CIs) in the equilibrium, initial and strain-dependent instantaneous moduli between the normal and moderate OA groups at medial and lateral femoral condyle cartilage

| | Parameter | Crude | Adjusted for age and BMI |
|---|---|---|---|
| Moderate OA vs normal, overall | $E_{eq}$ | -0.14 (-0.70, 0.42) | -0.01 (-0.61, 0.59) |
| | $E_{inst}^{0}$ | -2.31 (-6.20, 1.58) | -2.10 (-6.07, 1.87) |
| | $E_{\text{inst}}^{\varepsilon}$ | -2.39 (-25.94, 21.16) | -3.95 (-30.35, 22.45) |
| Moderate OA vs normal, medial compartment | $E_{eq}$ | -0.17 (-0.93, 0.60) | -0.05 (-0.87, 0.76) |
| | $E_{inst}^{0}$ | -6.69 (-13.08, -0.30) | -6.25 (-12.42, -0.07) |
| | $E_{\text{inst}}^{\varepsilon}$ | 3.62 (-33.26, 40.51) | 5.32 (-33.10, 43.74) |
| Moderate OA vs normal, lateral compartment | $E_{eq}$ | -0.11 (-0.72, 0.50) | 0.03 (-0.62, 0.68) |
| | $E_{inst}^{0}$ | 2.07 (-2.25, 6.39) | 2.05 (-2.19, 6.28) |
| | $E_{\text{inst}}^{\varepsilon}$ | -8.41 (-33.90, 17.09) | -13.22 (-41.23, 14.80) |

$E_{\text{eq}}$: Equilibrium modulus, $E_{\text{inst}}^{0}$: Initial instantaneous modulus, $E_{\text{inst}}^{\varepsilon}$ strain-dependent instantaneous modulus

## Detailed analyses of dynamic moduli and phase differences as a function of frequency

The raw dynamic moduli as a function of frequency for two OA status (i.e. normal and moderate OA) groups and locations (i.e. medial and lateral) are shown in figure S3.

Moreover, descriptive data for Elastic dynamic moduli measured at different frequencies are shown for each OARSI grade in Table S6.

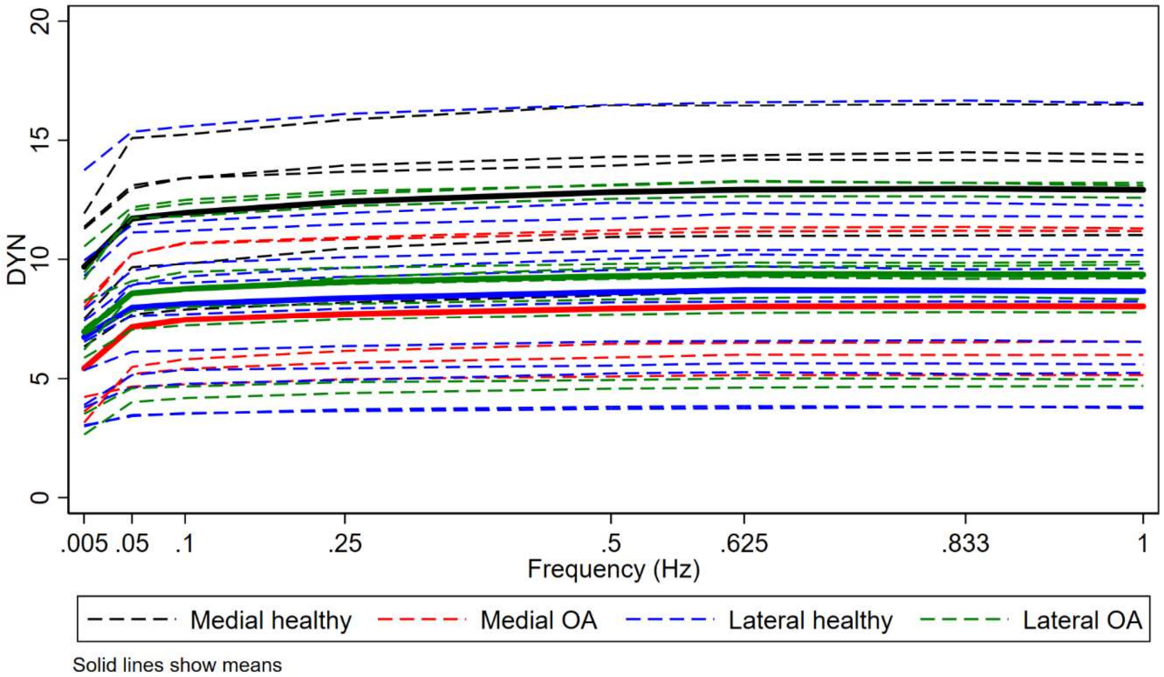


Figure S3: The dynamic moduli as a function of frequency. Solid lines show means.

Table S6: The dynamic elastic properties of human femoral condyle cartilage at different frequencies. The numbers are mean (SD) for medial, lateral and both compartments.

| Parameter | OARSI 0 (*n* = 5) | OARSI 1 (*n* = 12) | OARSI 2 (*n* = 12) | OARSI 3 (*n* = 3) | OARSI 4 (*n* = 3) |
|---|---|---|---|---|---|
| $E_{\text{dynamic}}^{0.005\,Hz}$ | 9.01 ± 3.87 | 7.01 ± 2.97 | 6.18 ± 2.55 | 7.54 ± 2.61 | 2.00 ± 1.26 |
| Med | 11.36 | 9.26 ± 2.75 | 5.43 ± 2.44 | - | 2.00 ± 1.26 |
| Lat | 8.43 ± 4.20 | 5.88 ± 2.51 | 6.71 ± 2.67 | 7.54 ± 2.61 | - |
| $E_{\text{dynamic}}^{0.05\,Hz}$ | 10.63 ± 4.12 | 8.43 ± 3.62 | 7.81 ± 2.93 | 9.27 ± 2.64 | 3.02 ± 1.48 |
| Med | 13.12 | 11.35 ± 3.31 | 7.15 ± 2.81 | - | 3.02 ± 1.48 |
| Lat | 10.00 ± 4.47 | 6.97 ± 2.93 | 8.28 ± 3.13 | 9.27 ± 2.64 | - |
| $E_{\text{dynamic}}^{0.1\,Hz}$ | 10.85 ± 4.13 | 8.60 ± 3.67 | 8.05 ± 2.99 | 9.47 ± 2.73 | 3.15 ± 1.57 |
| Med | 13.42 | 11.59 ± 3.33 | 7.47 ± 2.96 | - | 3.15 ± 1.57 |
| Lat | 10.21 ± 4.48 | 7.10 ± 2.95 | 8.45 ± 3.17 | 9.47 ± 2.73 | - |
| $E_{\text{dynamic}}^{0.25\,Hz}$ | 11.22 ± 4.28 | 8.88 ± 3.80 | 8.29 ± 3.03 | 9.87 ± 2.75 | 3.27 ± 1.61 |
| Med | 13.95 | 12.05 ± 3.39 | 7.71 ± 2.92 | - | 3.27 ± 1.61 |
| Lat | 10.53 ± 4.61 | 7.30 ± 3.03 | 8.71 ± 3.25 | 9.87 ± 2.75 | - |
| $E_{\text{dynamic}}^{0.5\,Hz}$ | 11.55 ± 4.32 | 9.16 ± 3.94 | 8.52 ± 3.09 | 10.14 ± 2.74 | 3.38 ± 1.62 |
| Med | 14.31 | 12.46 ± 3.47 | 7.94 ± 2.96 | - | 3.38 ± 1.62 |

| | | | | | |
|---|---|---|---|---|---|
| Lat | 10.86 ± 4.66 | 7.51 ± 3.14 | 8.93 ± 3.34 | 10.14 ± 2.74 | - |
| $E_{\text{dynamic}}^{0.625\ Hz}$ | 11.68 ± 4.33 | 9.23 ± 3.95 | 8.60 ± 3.11 | 10.24 ± 2.79 | 3.44 ± 1.64 |
| Med | 14.37 | 12.57 ± 3.45 | 8.03 ± 2.98 | - | 3.44 ± 1.64 |
| Lat | 11.00 ± 4.68 | 7.56 ± 3.14 | 9.01 ± 3.37 | 10.24 ± 2.79 | - |
| $E_{\text{dynamic}}^{0.833\ Hz}$ | 11.66 ± 4.40 | 9.24 ± 3.95 | 8.60 ± 3.10 | 10.24 ± 2.74 | 3.48 ± 1.65 |
| Med | 14.50 | 12.60 ± 3.45 | 8.04 ± 3.00 | - | 3.48 ± 1.65 |
| Lat | 10.96 ± 4.47 | 7.56 ± 3.13 | 9.00 ± 3.34 | 10.24 ± 2.74 | - |
| $E_{\text{dynamic}}^{1\ Hz}$ | 11.64 ± 4.33 | 9.20 ± 3.95 | 8.59 ± 3.09 | 10.22 ± 2.69 | 3.45 ± 1.64 |
| Med | 14.41 | 12.55 ± 3.44 | 8.03 ± 2.97 | - | 3.45 ± 1.64 |
| Lat | 10.95 ± 4.67 | 7.52 ± 3.12 | 8.99 ± 3.33 | 10.22 ± 2.69 | - |

Note that when medial and lateral compartments are pooled, the "standard deviation" is a combination of within and between subject variability.

The dynamic moduli were analyzed using three linear splines (with knots at 0.05 Hz and 0.5 Hz). The results of the linear mixed model for dynamic modulus are presented in Table S7.

Table S7: the differences (with 95%CIs) in dynamic modulus values between normal and moderate OA groups at medial and lateral femoral condyle cartilage.

| | Crude | Adjusted for age and BMI |
|---|---|---|
| Moderate OA vs normal, overall | -1.5 (-3.2, 0.3) | -1.3 (-3.2, 0.7) |
| Moderate OA vs normal, medial compartment | -1.8 (-4.5, 0.9) | -1.7 (-4.6, 1.2) |
| Moderate OA vs normal, lateral compartment | -1.1 (-3.0, 0.8) | -0.8 (-2.9, 1.3) |

The statistical model considers the person-specific effects (i.e. which samples are obtained from the same knees). To further clarify it, the dynamic moduli for each subject as a function of frequency is shown in figure S5. As can be seen, samples from the same person have similar dynamic moduli values irrespectively of moderate OA/normal status. Furthermore, dynamic moduli values are very similar in the medial and lateral compartments for all the samples. These findings showed that a strong person-specific component in the dynamic moduli values exists.

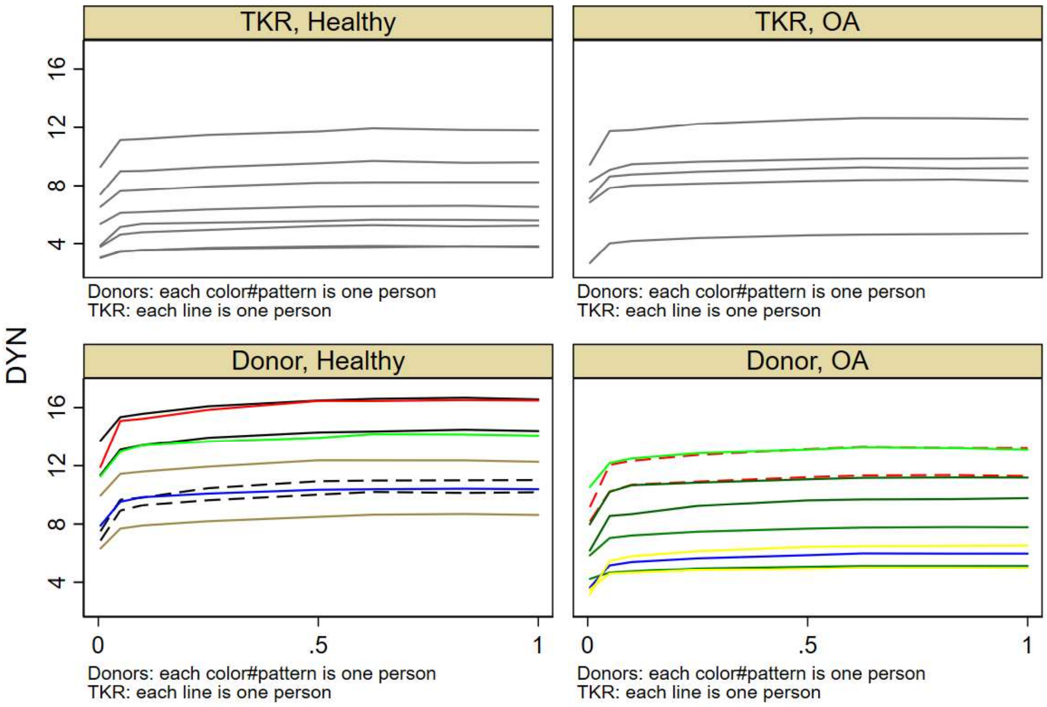


Figure S4: The dynamic moduli values for each subject shown as a function of frequency.

The raw phase differences as a function of frequency for two groups (normal and moderate OA) groups and compartments (medial and lateral) are shown in figure 3.

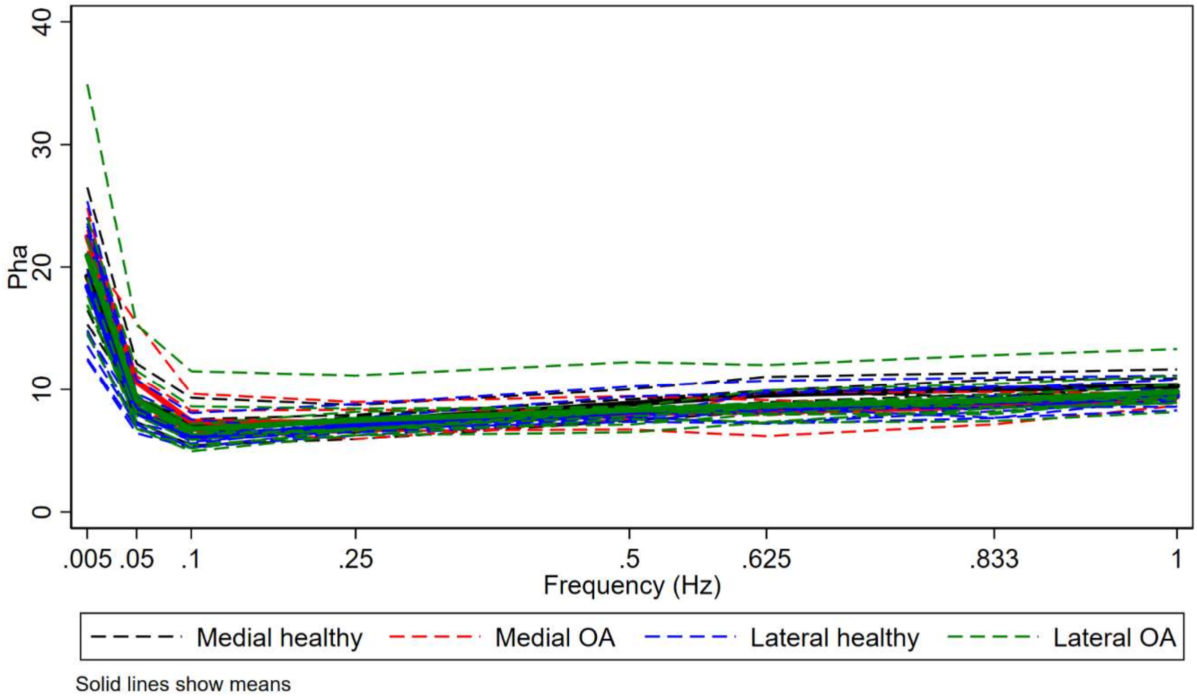


Figure S5: The phase differences values for each subject shown as a function of frequency. Solid lines represent the means.

Moreover, descriptive data for dynamic viscoelastic properties (i.e. phase difference) measured at different frequencies are shown for each OARSI grade in Table S8.

Table S8: The dynamic viscoelastic properties (phase difference) of human femoral condyle cartilage. The numbers are mean (SD) for medial, lateral and both compartments.

| Parameter | OARSI 0 (*n* = 5) | OARSI 1 (*n* = 12) | OARSI 2 (*n* = 12) | OARSI 3 (*n* = 3) | OARSI 4 (*n* = 3) |
|---|---|---|---|---|---|
| $\theta_{\text{dynamic}}^{0.005\,Hz}$ | 19.98 ± 5.22 | 18.10 ± 4.74 | 21.34 ± 5.34 | 21.84 ± 2.84 | 29.34 ± 6.13 |
| Med | 16.47 | 19.96 ± 4.76 | 22.43 ± 2.35 | - | 29.34 ± 6.13 |
| Lat | 20.86 ± 5.58 | 17.18 ± 4.77 | 20.55 ± 6.84 | 21.84 ± 2.84 | - |
| $\theta_{\text{dynamic}}^{0.05\,Hz}$ | 8.77 ± 1.50 | 8.29 ± 1.53 | 9.61 ± 2.29 | 9.66 ± 1.84 | 16.46 ± 5.20 |
| Med | 8.36 | 9.35 ± 1.99 | 10.63 ± 2.86 | - | 16.46 ± 5.20 |
| Lat | 8.87 ± 1.71 | 7.76 ± 1.01 | 8.88 ± 2.93 | 9.66 ± 1.84 | - |
| $\theta_{\text{dynamic}}^{0.1\,Hz}$ | 6.68 ± 1.22 | 6.26 ± 1.19 | 6.91 ± 1.95 | 7.22 ± 1.28 | 11.01 ± 3.66 |
| Med | 6.55 | 7.26 ± 1.63 | 7.37 ± 1.55 | - | 11.01 ± 3.66 |
| Lat | 6.71 ± 1.28 | 5.76 ± 0.47 | 6.59 ± 2.24 | 7.22 ± 1.28 | - |
| $\theta_{\text{dynamic}}^{0.25\,Hz}$ | 7.38 ± 0.93 | 7.00 ± 0.78 | 7.34 ± 1.50 | 7.81 ± 0.86 | 10.60 ± 3.28 |
| Med | 7.80 | 7.27 ± 1.29 | 7.29 ± 1.29 | - | 10.60 ± 3.28 |
| Lat | 7.27 ± 1.03 | 6.87 ± 0.43 | 7.37 ± 1.73 | 7.81 ± 0.86 | - |
| $\theta_{\text{dynamic}}^{0.5\,Hz}$ | 8.52 ± 1.20 | 8.30 ± 0.82 | 8.21 ± 1.50 | 8.32 ± 0.45 | 11.12 ± 2.41 |
| Med | 9.20 | 8.72 ± 1.11 | 8.06 ± 0.99 | - | 11.12 ± 2.41 |
| Lat | 8.34 ± 1.31 | 8.08 ± 0.60 | 8.31 ± 1.85 | 8.32 ± 0.45 | - |
| $\theta_{\text{dynamic}}^{0.625\,Hz}$ | 9.25 ± 1.35 | 8.68 ± 1.09 | 8.37 ± 1.46 | 9.10 ± 0.75 | 11.31 ± 2.24 |
| Med | 9.75 | 9.52 ± 1.22 | 8.20 ± 1.36 | - | 11.31 ± 2.24 |
| Lat | 9.12 ± 1.52 | 8.25 ± 0.79 | 8.49 ± 1.62 | 9.10 ± 0.75 | - |
| $\theta_{\text{dynamic}}^{0.833\,Hz}$ | 9.60 ± 1.05 | 9.17 ± 1.14 | 8.77 ± 1.44 | 9.68 ± 0.72 | 11.12 ± 2.04 |
| Med | 9.40 | 10.18 ± 1.08 | 8.60 ± 0.94 | - | 11.12 ± 2.04 |
| Lat | 9.64 ± 1.21 | 8.67 ± 0.82 | 8.89 ± 1.78 | 9.68 ± 0.72 | - |
| $\theta_{\text{Dynamic}}^{1\,Hz}$ | 10.05 ± 8.87 | 9.62 ± 1.00 | 9.51 ± 1.29 | 10.31 ± 0.87 | 12.12 ± 2.40 |
| Med | 10.39 | 10.26 ± 1.25 | 9.40 ± 0.55 | - | 12.12 ± 2.40 |
| Lat | 8.96 ± 0.87 | 9.29 ± 0.74 | 9.58 ± 1.67 | 10.31 ± 0.87 | - |

Note that when medial and lateral compartments are pooled, the “standard deviation” is a combination of within and between subject variability.

The mean difference and 95% CI in phase difference values between normal and moderate OA groups at medial and lateral femoral condyle cartilage can be seen in Table S9.

Table S9: The differences (with 95%CIs) in the phase difference values between the normal and moderate OA groups at medial and lateral femoral condyle cartilage.

| | Freq. (Hz) | Crude | Adjusted for aga and BMI |
|---|---|---|---|
| Moderate OA vs normal, overall | 0.005 | 2.91 (-0.58, 6.41) | 3.02 (-0.49, 6.54) |
| | 0.05 | 1.29 (0.07, 2.50) | 1.40 (0.11, 2.69) |
| | 0.1 | 0.53 (-0.68, 1.74) | 0.64 (-0.65, 1.93) |
| | >=0.25 | -0.28 (-1.30, 0.74) | -0.17 (-1.28, 0.94) |
| Moderate OA vs normal, medial compartment | 0.005 | 3.20 (-2.35, 8.75) | 3.36 (-2.21, 8.93) |
| | 0.05 | 1.48 (-0.42, 3.39) | 1.65 (-0.32, 3.62) |
| | 0.1 | 0.26 (-1.65, 2.17) | 0.42 (-1.55, 2.39) |
| | >=0.25 | -0.90 (-2.44, 0.64) | -0.74 (-2.36, 0.88) |
| Moderate OA vs normal, lateral compartment | 0.005 | 2.63 (-1.13, 6.39) | 2.69 (-1.09, 6.47) |
| | 0.05 | 1.09 (-0.22, 2.39) | 1.15 (-0.21, 2.50) |
| | 0.1 | 0.80 (-0.51, 2.10) | 0.85 (-0.50, 2.21) |
| | >=0.25 | 0.34 (-0.72, 1.40) | 0.40 (-0.73, 1.52) |

Similar to dynamic moduli, a strong person-specific effect exists. The statistical model considers the person-specific effects (i.e. which samples are obtained from the same knees). To further clarify it, the phase differences for each subject as a function of frequency are shown in figure S6.

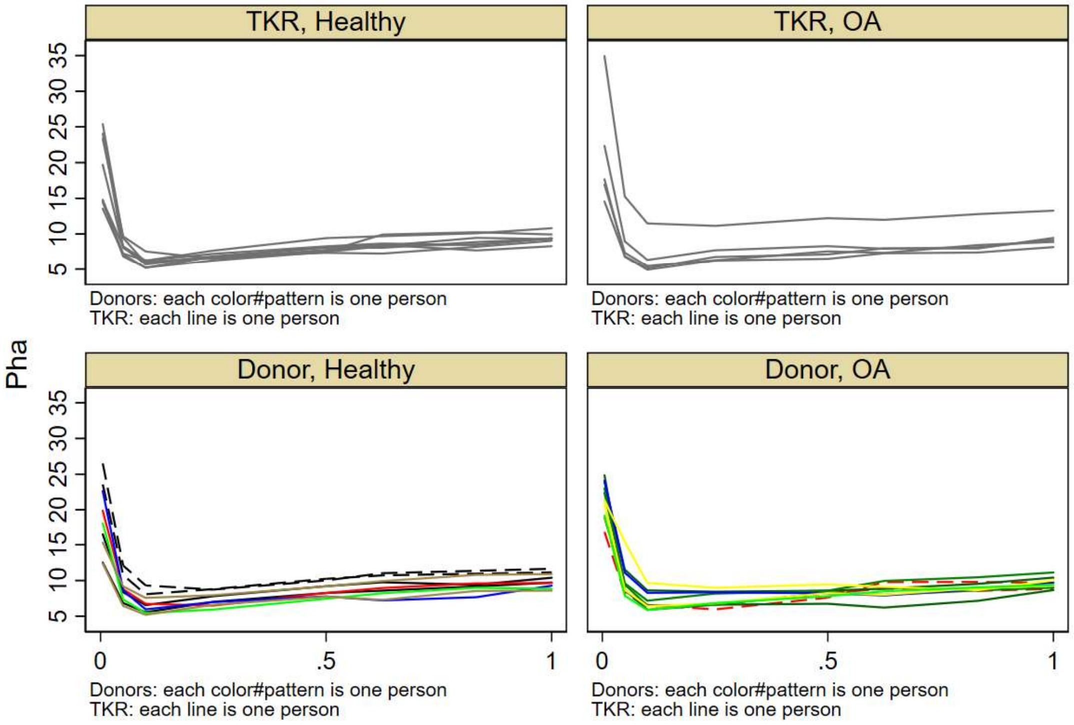


Figure S6: The phase differences values for each subject shown as a function of frequency.
